# HYNIC and DOMA conjugated radiolabeled bombesin analogs as receptor-targeted probes for scintigraphic detection of breast tumor

**DOI:** 10.1186/s13550-019-0493-x

**Published:** 2019-03-18

**Authors:** Kakali De, Dibyanti Mukherjee, Samarendu Sinha, Shantanu Ganguly

**Affiliations:** 1 Infectious Diseases and Immunology Division (Nuclear Medicine Laboratory), CSIR-Indian Institute of Chemical Biology, 4 Raja S. C. Mullick Road, Kolkata, West Bengal 700032 India; 2 Regional Radiation Medicine Center, Thakurpukur Cancer Research Center and Welfare Home Campus, Kolkata, West Bengal 700 060 India

**Keywords:** Bombesin, Binding affinity, GRP receptor, Scintigraphy image

## Abstract

**Background:**

Among the many peptide receptor systems, gastrin-releasing-peptide (GRP) receptors, the mammalian equivalent of bombesin (BN) receptors, are potential targets for diagnosis and therapy of breast tumors due to their overexpression in various frequently occurring human cancers. The aim of this study was to synthesize and comparative evaluation of ^99m^Tc-labeled new BN peptide analogs. Four new BN analogs, HYNIC-Asp[PheNle]BN(7-14)NH_2_, BN1; HYNIC-Pro-Asp[TyrMet]BN(7-14)NH_2_, BN2; HYNIC-Asp-Asn[Lys-CHAla-Nle]BN(7-14)NH_2_, BN3; and DOMA-GABA[Pro-Tyr-Nle]BN(7-14)NH_2_, BN4 were synthesized and biologically evaluated for targeted imaging of GRP receptor-positive breast-tumors.

**Methods:**

Solid-phase synthesis using Fmoc-chemistry was adopted for the synthesis of peptides. BN1–BN4 analogs were better over the standard Gln-Trp-Ala-Val-Gly-His-Leu-Met-NH_2_ (BNS). Lipophilicity, serum stability, internalization, and binding affinity studies were carried out using ^99m^Tc-labeled analogs. Biodistribution and imaging analyses were performed on MDA-MB-231 cell-induced tumor-bearing mice. BN-analogs induced angiogenesis; tumor formation and GRP-receptor-expression were confirmed by histology and immunohistochemistry analyses of tumor sections and important tissue sections.

**Results:**

All the analogs displayed ≥ 97% purity after the HPLC purification. BN-peptide-conjugates exhibited high serum stability and significant binding affinity to GRP-positive tumor; rapid internalization/externalization in/from MDA-MB-231 cells were noticed for the BN analogs. BN4 and BN3 exhibited higher binding affinity, stability than BN1 and BN2. Highly specific in vivo uptakes to the tumor were clearly visualized by scintigraphy; rapid excretion from non-target tissues via kidneys suggests a higher tumor-to-background ratio. BN4, among all the analogs, stimulates the expression of angiogenic markers to a maximum.

**Conclusion:**

Considering its most improved pharmacological characteristics, BN4 is thus considered as most promising probes for early non-invasive diagnosis of GRP receptor-positive breast tumors.

**Electronic supplementary material:**

The online version of this article (10.1186/s13550-019-0493-x) contains supplementary material, which is available to authorized users.

## Background

Studies involving the design and development of peptide-based diagnostic radiopharmaceuticals for the early detection of human cancer are in ascendance [1–4] that helped in reducing the number of cancerous deaths. Small peptides can penetrate into tumor tissues promoting rapid blood clearance than other macromolecules [5, 6]*.* Gastrin-releasing peptide receptor (GRPR) is overexpressed in high incidence among the different types of human cancerous tumors (prostate, breast, gastrointestinal stromal, and lung cancer) [1–8]. Tailor-made positive charged radiolabeled GRPR are one of the ideal molecular targets in diagnosing breast cancer. Breast cancer is common among women and is the second most frequent cause of cancer-related deaths worldwide [6, 7]. Bombesin (BN) (pharmacological homologs of GRP) is a 14-amino acid amphibian peptide homolog of 27-amino acids, with resemblances to the mammalian gastrin-releasing peptides (GRP). GRP/BN promotes breast cancer growth and progression [4, 5]; the occurrence of these high-affinity receptors in breast cancer has instigated the scientists to develop newer BN-based diagnostic agents [6, 7]. They share a homologous 7-amino acid amidated C-terminus, Gln-Trp-Ala-Val-Gly-His-Leu-Met-NH_2_ (BNS), necessary for binding to GRPR. Due to poor in vivo stability of the native GRPRs, different BN analogs have been developed for biological activity studies. BN like peptides exert their effects on target cells by binding to surface G protein-coupled receptors. For synthetic truncated bombesin, eight carboxy-terminal residues [BN(7-14)NH_2_] are kept intact to retain its affinity for GRP receptor, necessary in identifying the tumors. C-terminus of BN possesses high affinity binding and biological potency [6–10]; thus the N-terminus of BN analogs are usually modified for labeling with radioisotopes that have significant potentials as targeting agents for preoperative tumor localization, assessment of lymph node involvement, staging of diseases and therapeutic monitoring of cancers, etc. [4–14]. High tumor uptake, with negligible uptakes in non-targeted tissues, is one of the crucial criteria in developing peptide-based radiopharmaceuticals. Rapid pharmacokinetics is ideal for labeling peptides with the radioisotopes having a short half life time. Among all the radioisotopes used in nuclear medicine, technetium-99m (^99m^Tc) is still the most widely used radioisotope for diagnostic purposes, because of its ease of availability, low cost, excellent imaging properties, favorable dosimetry, and high specific activity, to mention a few. Thus the development of newer BN peptide radiopharmaceuticals with improved stability and high GRP receptor binding affinity are warranted in obtaining a more homogeneous distribution of the activity within the tumor mass for early diagnosis of cancer.

While radiolabeling BN(7-14)NH_2_ with ^99m^Tc, different bifunctional chelating ligands, like hydrazinonicotinic acid (HYNIC), 1,4,7,10-tetraazacyclododecane-1,4,7,10-tetraacetic acid (DOTA), 1,4,7-triazacyclononane-1,4,7-triacetic acid (NOTA), and *N*,*N*′,*N*″,*N*‴-tetrakis(*tert*-butyloxycarbonyl)-6-(carboxy)-1,4,8,11-tetra-azaundecane (N4), are considered as good chelating ligands as the carboxylic acid groups can react with the N-termini of peptides. It does not affect BN(7-14)NH_2_-GRPR binding process, as it occurs near the C-terminal residue. Besides, the inclusion of additional amino acids as spacers are also required in between the ligand and BN(7-14)NH_2_ without compromising its interaction capabilities with different receptors. Also the addition of the co-ligands like tricine and ethylene diamine diacetic acid (EDDA), in the radiolabeling procedure of ligand conjugated BN(7-14)NH_2_, stabilizes the metal complex [10–18]. Therefore, a successful candidate that shows superior pharmacokinetics and tracer accumulation in cancerous tumor with ^99m^Tc-labeled BN analogs is considered interesting for cancer diagnosis [19–23]. HYNIC/DOMA (1,4,7-tri-Boc-10-(carboxymethyl)-1,4,7,10-tetraazocyclododecane-1-yl-monoacetic acid), a bifunctional chelating agent, was conjugated with new BN peptide analogs, HYNIC-Asp-[d-Phe^13^Nle^14^]BN(7-14)NH_2_,BN1; HYNIC-Pro-Asp-[d-Tyr^13^Met^14^] BN(7-14)NH_2_, BN2; HYNIC-Asp-Asn-[Lys^5^-d-CHAla^13^Nle^14^]BN(7-14)-NH_2_,BN3; and DOMA-GABA-[Pro^5^-d-Tyr^13^Nle^14^]-BN(7-14)-NH_2_, BN4, radiolabeled with ^99m^Tc. Biological activity studies were carried out with the expectations of improved metabolic stability and binding affinity to GRP receptor-positive tumor for targeted imaging studies. Vis-à-vis comparison was made with the standard, BN(7-14)NH_2_ (henceforth to be termed as BNS). To increase the stability, some structural modifications at the C-terminus of the BNS amino acid sequence (exchange of Met at position 14 with non-natural amino acid norleucine (Nle) and Leu at position 13 with non-natural amino acid cyclohexylalanine (CHAla)/d-Tyr and Gly at position 5 with d-Lys/d-Pro, respectively, were made in the newly synthesized analogs. It is expected that the newly derived analogs will have improved stability in plasma and in breast cancer cells. Increase in the hydrophilicity, as well as the presence of ionic charges are expected to improve biodistribution profiles; thus spacers such as Asp/Asn/Pro/GABA amino acid were also inserted between the chelator and the BN sequences. Receptor binding affinity, internalization profile in GRP receptor-positive MDA-MB-231 breast cancer cell lines was studied. Biodistribution studies of the new analogs, with respect to the GRP-positive tumor xenografted in mice, as well as the scintigraphy studies were performed. Moreover, attempts were made to show that BN analogs treatment could enhance tumor growth and expression of angiogenic markers. It is believed that the present set of studies could assess the feasibility of new BN analogs as improved imaging probes for the early diagnosis of breast cancer.

## Materials and methods

### Reagents and instrumentation

Rink amide MBHA resin (100–200 mesh), all 9-fluorenylmethyloxycarbonyl (Fmoc)-amino acid and TBTU [2-(1H-benzotriazol-1-yl)-1,1,3,3-tetramethyluronium tetramethyluronium tetrafluoroborate], was purchased from Nova Biochem (San Diego, CA, USA). Diisopropylethylamine (DIPEA), thioanisole, piperidine, dimethyl formamide (DMF), ethylene diamine diacetic acid (EDDA), tricine, SnCl_2_, and picryl sulfonic acid were purchased from Sigma-Aldrich Chemicals, USA. Electronics Corporation of India Ltd. (ECIL) gamma counter (Model L V4755) was used for the radioactivity quantification. The cell culture medium was Dulbecco’s modified Eagle’s medium (DMEM) supplemented with 10% fetal bovine serum (FBS); amino acids, vitamins, and penicillin/streptomycin from Gibco, USA (sodium ^99m^Tc-pertechnetate [Na(^99m^TcO_4_)] were obtained by 2-butanone extraction from a 5(N) NaOH solution of ^99^MoO_4_^−^ (Bhaba Atomic Research Centre, Mumbai, India) and technetium generator (obtained from the CSIR-Indian Institute of Chemical Biology) was used for radiopharmaceutical preparation. Analytical and preparative HPLC studies were carried out on a WATERs RP-HPLC system equipped with *μ* bondapak C18, 10 μm, 125 A, 7.8 × 300 mm reverse-phase column and UV detector (WATERS, USA), set at 254 nm, using gradient solvent systems. Berthold LB500 HERM radio HPLC monitor (Berthold Technologies) was used to analyze radioactivity in the HPLC elutes. MDA-MB-231 human breast cancer cell line, obtained from NCCS, Pune, India was used for cancer cell line studies. Human GRP receptor-positive cell membranes were purchased from Millipore, Inc. (Billerica, MA, USA) for receptor binding studies. GRPR antibody was from Abcam, Inc. USA for immunohistochemistry (IHC) staining. Horseradish peroxidase (HRP)-conjugated secondary antibodies were purchased from Santa Cruz Biotechnology Inc. (Dallas, USA). All other chemicals were purchased from Thermo Fischer Scientific (Waltham, MA, USA) and were used without further purification.

### Synthesis of peptide analogs

The pro-chelator Boc-HYNIC was synthesized according to the standard method [24–26]. The bombesin peptides were synthesized using 9-fluorenylmethyloxycarbonyl (Fmoc) chemistry on solid support [24–27]. Briefly, HYNIC-Asp-(Gln-Trp-Ala-Val-Gly-His-Phe-Nle)-NH_2_: BN1;HYNIC-Pro-Asp-(Gln-Trp-Ala-Val-Gly-His-Tyr-Met)-NH_2_: BN2; HYNIC-Asp-Asn-(Gln-Trp-Ala-Val-Lys-His-ChAla-Nle)-NH_2_: BN3; and DOMA-GABA- (Gln-Trp-Ala-Val-Pro-His-Tyr-Nle)-NH_2_: BN4 peptides were assembled on Rink amide MBHA resin by the manual process using Fmoc-protected amino acids with appropriate side-chain protection. Then, 0.1 mmol resin was treated with 20% (*V*/*V*) piperidine in DMF under constant nitrogen purging (15 min) followed by repeated washing with DMF to remove the Fmoc group. Coupling of each amino acid to the deprotected resin (placed in a small glass column under nitrogen purging) was performed in the presence of 5 mol excess of Fmoc-amino acid (0.5 mmol), 0.5 mmol TBTU, and 1 mmol DIPEA, in DMF (3 mL). The complete synthesis was carried out by stepwise coupling of Fmoc-amino acids to the growing peptide chain on the resin [4, 24–27]. This process was repeated until the required sequence was achieved. Finally, a coupling of pro-chelator to the peptide for BN1-BN3 was performed in presence of 5 mol excess of BOC-HYNIC chelator and in the case of BN4, macrocyclic chelator DOMA (1,4,7-tri-Boc-10-(carboxymethyl)-1,4,7,10-tetraazocyclododecane-1-yl)-acetic acid) along with 4.5 mol excess of TBTU and 10 mol excess of DIPEA in DMF for 40 min. Coupling success was checked by the TNBS test. The coupled resin was filtered and rinsed with DMF. The average coupling yield was calculated from the increase in weight of the elongated peptide resin divided by the weight of protected peptide. The HYNIC/DOMA conjugated BN analogs with all protecting groups was cleaved after treatment with a cocktail of TFA:triisopropylsilane (TIS):water (95:2.5:2.5, *V*/*V*/*V*) at 25 °C for 2–3 h. After removing the organic solvent under vacuum, the crude products of BN1-BN4 were separately precipitated with cold diethyl ether and washed with ice-cold ether for four times. BN peptides were finally purified by semi-preparative reverse phase-HPLC (RP-HPLC). Injections of 200 μL were eluted at a flow rate of 2 mL/min from a *μ* bondapack C-18, reverse-phase column, using a 32-min binary gradient system consisting of 0.1% TFA in water (solvent A) and 0.085% TFA in acetonitrile (solvent B). Gradient I: 0 min 80% A (20% B), 2 min 80% A (20% B), 17 min 50% A (50%B), 19 min 0% A (100% B), 21 min 0% A (100% B), 24 min 80% A (20% B), 32 min 80% A (20%B). The column effluent was monitored using a UV detector set at 280 nm. The major isolated peaks for all the peptides were collected, after which they were lyophilized and stored at − 20 °C. Finally, the purified HYNIC-Asp-[d-Phe^13^Nle^14^]BN(7-14)-NH_2_, HYNIC-Pro-Asp-[d-Tyr^13^Met^14^]BN(7-14)-NH_2_, HYNIC-Asp-Asn-[Lys^5^-d-CHAla^13^Nle^14^]BN(7-14)-NH_2_, and DOMA-GABA-[Pro^5^-d-Tyr^13^Nle^14^]BN(7-14)-NH_2_ were analyzed by matrix-assisted laser desorption ionization (MALDI) spectrometry in positive-ion mode and analytical HPLC. In the same method, Gln-Trp-Ala-Val-Gly-His-Leu-Met)-NH_2_ (standard, BNS) have been synthesized, purified, and used as a standard for comparison of these new BN analogs.

### Radiolabeling and purification of labeled peptide conjugates for biological studies

Radiolabeling of HYNIC conjugate peptides BN1, BN2, and BN3 and DOMA conjugated BN4 with ^99m^Tc was done by standard procedure, as reported earlier [24–26]. Nitrogen purging, prior to mixing, was carried out to degas all the solutions. The stock solution of the peptide analogs (1 mM) was prepared by dissolving it in distilled water. Then, 20 μL stock solution, 20 mg tricine, and 10 mg EDDA co-ligands in 0.5 mL water were added into a 5 mL Eppendorf tube. Freshly prepared stannous chloride solution (SnCl_2_.2H_2_O, 25 μg) was added in this solution. Finally, ^99m^Tc (37–370 MBq) in 0.5 mL saline was added to this solution and incubated for 15 min at 100 °C. Final pH of the labeling solution was adjusted to 7–8. After incubation and subsequent cooling to room temperature, radiochemical purity assessment and quality control of the labeled peptide was achieved by RP-HPLC and instant thin-layer chromatography on silica gel plates (ITLC-SG). The mobile phase used in RP-HPLC for gradient system consisted of 0.1% TFA/water (solvent A), 0.85% TFA/acetonitrile (solvent B) at 1 mL/min flow rate. Gradient II: 0 min 95% A (5% B), 5 min 95% A (5% B), 25 min 0% A (100% B), 27 min 0% A (100% B), 30 min 95% A (5% B), 35 min 100% A (0% B). ITLC-SG was performed using different mobile phases. 2-Butanone was used to determine the amount of free ^99m^TcO_4_^¯^ (*R*_f_ = 1.0), 0.1 M sodium citrate of pH 5 was used to determine the non-peptide bound ^99m^Tc-coligand and ^99m^TcO_4_^¯^ (*R*_f_ = 1), and methanol/1 M ammonium acetate (1/1) was used for ^99m^Tc-colloid (*R*_*f*_ = 0.0). In the same way, BNS was radiolabeled, purified, and was used as a standard.

### In vitro stability studies

In vitro radiochemical stability of ^99m^Tc-BN1/^99m^Tc-BN2/^99m^Tc-BN3 and ^99m^Tc-BN4, ^99m^Tc-BNS were assessed by incubating HPLC purified radiopeptides in phosphate buffer saline (PBS at pH 7.4) and freshly collected rat serum for 1–24 h at room temperature [24–26]. Then, 90 μL of radiolabeled peptide ^99m^Tc-BN1/^99m^Tc-BN2/^99m^Tc-BN3/^99m^Tc-BN4/^99m^Tc-BNS solution were incubated under agitation at 37 °C with either 1.0 mL saline or fresh rat serum, and the mixtures were incubated at 37 °C for 24 h. Samples were periodically withdrawn from the mixture at 0, 2, 4, 6, 8, 12, and 24 h incubation period and analyzed by ITLC. In the case of rat serum, the aliquot was added to 100 μL 50% trifluoroacetic acid (TFA). After centrifugation (3000 rpm), the supernatant was analyzed by ITLC using acetone as the developing solvent; 10 μL of this mixture was taken at each time point, added to a strip ITLC whereby the percentage of ^99m^Tc-BN1/^99m^Tc-BN2/^99m^Tc-BN3/^99m^Tc-BN4/^99m^Tc-BNS was determined using gamma counter.

### Cysteine and histidine challenge

Synthesized radiolabeled BN analogs were tested for instability toward cysteine and histidine. A fresh known amount of ^99m^Tc-BN1/^99m^Tc-BN2/^99m^Tc-BN3/^99m^Tc-BN4/^99m^Tc-BNS was incubated with an excess of cysteine or histidine (100-fold molar excess compared to the peptide) at 37 °C for 4 h. After incubation, the percentage of radioactivity associated with cysteine or histidine and the intact radiolabeled peptide was determined by ITLC [24–26].

### Octanol–buffer partition coefficient

Lipophilicity of the HPLC-purified radiopeptide analogs was determined through octanol/water partition coefficients, determined at pH 7.4 by measuring the distribution of the HPLC purified radiolabeled peptides in 1-octanol and PBS. Then, 50 μL HPLC purified ^99m^Tc-BN1/^99m^Tc-BN2/^99m^Tc-BN3/^99m^Tc-BN4/^99m^Tc-BNS with 100 μCi of radiolabeled compound in PBS was added to a to a centrifuge tube containing 1 mL *n*-octanol and 1 mL PBS (0.025 M, pH 7.4). The tube was vortexed at room temperature for 2 min and they are centrifuged at 5000 rpm for 5 min to ensure complete separation of layers. Further, 100 μL of each phase was transferred into a separate pre-weighed vial and measured in the well γ-counter. The ratio of octanol layer counts to aqueous layer counts was expressed as the distribution coefficient (*D*, the appropriate descriptor for ionizable compounds) and *logD* was used for determination of lipophilicity. Counts per unit weight of the sample were calculated, and *logD* value as calculated as follows:

*log*_10_*D* = *log*_10_ [(cpm in octanol – cpm in background) / (cpm in buffer – cpm in background)]

### Plasma protein binding assay

An aliquot (100 μL) of ^99m^Tc-BN1/^99m^Tc-BN2/^99m^Tc-BN3/^99m^Tc-BN4/^99m^Tc-BNS was added to 1 mL of freshly collected rat serum in centrifuge tube. The mixture was then incubated at 37 °C for 1 h; the serum protein was precipitated by adding 1 mL trichloroacetic acid (10%, *V*/*V*). The supernatant and precipitate were separated by centrifugation at 3000 rpm for 5 min. Radioactivities of both the phases were measured separately. The above experimental procedure was repeated three times. The percentage of protein binding was determined as [4, 24–27]:

Plasma protein binding % = (precipitate counts (CPM) / [precipitate counts (CPM) + plasma counts (CPM)]) × 100%.

### Blood clearance studies

^99m^Tc-labeled HYNIC/DOMA conjugated peptide analogs (1 mCi, 0.2 mL) were administered to well-hydrated mouse (*n* = 3) through tail vein and blood samples (20 μL each) were collected at preset time intervals (2, 5, 15, 30, 60, 90, and 120 min) in the tube. Each sample was weighed accurately, and associated radioactivity was counted in a gamma counter [4, 24–27]. The percentage injected activities per gram (%IA/g) in each blood sample at each time were determined, and the data were plotted as a function of the time interval to obtain the blood clearance curve of the radiopharmaceuticals.

### Cell culture condition

MDA-MB-231 human breast cancer cell line was procured from the National Centre for Cell Science (NCCS), Pune, India. This cell line was maintained in Dulbecco’s modified Eagle’s medium (DMEM) supplemented with 10% (*V*/*V*) fetal bovine serum (FBS) and antibiotics (penicillin and streptomycin obtained from Gibco, Mumbai, India). Cells were kept in T-25 tissue culture flask in humidified air containing 5% CO_2_ at 37 °C. The cells were grown to confluence and later harvested by trypsinization. After centrifugation (1600 rpm, 5 min), cells were re-suspended in PBS for inoculation into the mice [24–26].

### Stability in MDA-MB-231 cell culture medium

Stability of the ^99m^Tc-BN analogs, the cell culture medium was examined by incubating the radiopeptides (40 μL, 30 μCi) with cell culture medium at 37 °C. Aliquots were sampled by TLC using silica gel TLC strips at appropriate times (0, 1, 3, 24 h) with a mobile phase of 5% 6 N HCl in methanol. Radioactivity distribution on the TLC strip was measured by well γ-counter [24–27].

### In vitro receptor binding assay

One day prior to the assay, cells at the confluence were placed in 12-well plates. The in vitro GRPR binding affinity assays were performed with synthesized analogs on MDA-MB-231 cells in a competitive binding assay using ^99m^Tc-BNS as the radioligand. Inhibitory assays were done on GRPR-expressing MDA-MB-231 human breast cancer cells using the standard procedure [4, 24–27] with modifications. One day prior to the assay, cells at the confluence were placed in 12-well plates. Cells were incubated for 1 h at 37 °C in a binding buffer (50 mM HEPES, 125 mM NaCl, 7.5 mM KCl, 5.5 mM MgCl_2_.6H_2_O, 1 mM EGTA, 5 g/L BSA, 50 mg/L bacitracin, pH 7.4) with increasing concentration of the unlabeled BN analogs (0–30,000 nM) in the presence of 4 kBq of ^99m^Tc-BNS per well, which is known to express a binding affinity to the GRP receptor. After 1-h incubation, cells were washed twice with cold PBS. Receptor bound radioactivity was recovered by solubilizing the cells with 400 μL 1 N NaOH at 37 °C. Binding activities were evaluated in the gamma counter. IC_50_ (the best-fit 50% inhibitory concentration) values were determined using nonlinear regression.

### Cell uptake and washout studies

Cell uptake (internalization) and washout (externalization) studies of all the four analogs, as well as the standard, were performed with MDA-MB-231 cells. Two days prior to the assay, MDA-MB-231 cells at confluence were placed in 6-well plates at a density of 1 × 10^5^ cells per well and maintained. After incubation, cells were washed three times with PBS and then 1 mL internalization medium was added to each well, followed by the addition of 150 μL ^99m^Tc-BN analogs in 1% (*w*/*v*) BSA/PBS buffer and either 150 μL of 1% (*W*/*V*) BSA/PBS buffer (to measure total counts) or 150 μL of 10 μM competitor (BNS) in 1% (*W*/*V*) BSA/PBS buffer (to measure the nonspecific binding). The wells corresponding to total binding and nonspecific binding were pipette in triplicate for each time point of 2, 5, 15, 30, 60, and 120 min of incubation at 37 °C. Incubation was stopped after the desired time interval by removing the medium and washing the cells twice with ice cold PBS. Receptor-bound radioligand was removed; an acid wash was carried out twice with 0.1 M glycine buffer pH 2.8 for 10 min on ice and placed in counting tubes. For internalization analysis, the cells were then lysed with 1 M NaOH, which was removed to counting tubes after 15 min, along with 1 × 1 mL of PBS wash (the internalized fraction). Radioactivity of the culture medium, the receptor-bound, and the internalized fractions was measured with the gamma counter. After counting the tubes, the specific internalized mean values were calculated and represented as a percentage of total activity added. Experiments were repeated thrice and the results were expressed as the mean ± standard deviation (SD).

In the washout experiment, MDA-MB-231 cells were seeded into 6-well plates at a density of 1 × 10^5^ cells per well before overnight incubation. After incubation, the cells were rinsed three times with PBS and then ^99m^Tc-BN1/^99m^Tc-BN2/^99m^Tc-BN3/^99m^Tc-BN4 peptide was added in triplicate. After 120-min incubation at 37 °C, the cells were washed with PBS, and then re-incubated in serum-free medium. The cells were washed with PBS and lysed with 0.1 M NaOH at 10, 20, 30, 60, 90, 120, 180, and 240 min interval. The cell lysate was collected and the remaining radioactivity was measured with the gamma counter. Cell uptake and washout values were normalized in terms of added radioactivity and expressed as the percent added radioactivity.

### Animals

Animal studies were conducted using female BALB/c mice (3–4 weeks of age) weighing 50–60 g housed under aseptic conditions, which included filtered air and sterilized food, water, bedding, and cages. All the experiments were performed in compliance with the approval of the Institutional Animal Ethics Committee of the CSIR-Indian Institute of Chemical Biology, Kolkata, India.

### Tumor model development

All animal experiments were done according to the CSIR-IICB Animal Ethics Committee’s rules and regulation. Forty-eight hours before tumor cell implantation, an immunosuppressive drug (cyclosporine-A) was given intraperitoneal (i.p) at a concentration of 30 mg/kg/day. After that, MDA-MB-231 cells (5 × 10^6^) 0.2 mL were implanted subcutaneously into the left flanks of the hind leg of BALB/c mice [26, 28–30]. Thereafter, the same dose of cyclosporine was given i.p. daily up to 2 weeks. Two weeks after inoculation of the tumor cells, when tumors reached ~ 0.5 cm in mean diameter, the tumor-bearing mice were used for biodistribution and imaging studies. Animals were monitored daily and their weights were recorded. Control group of mice received only 0.9 wt% saline. After cell implantation, the growth rate of the breast tumor followed a satisfactory pattern of spheroid shape and tumors are palpable within 2 to 4 weeks. MDA-MB-231 cell implanted mice tumor models were used for biodistribution and planar imaging studies of these radiolabeled new bombesin analogs.

### Histopathological analysis

After performing biodistribution studies, MDA-MB-231 cell-induced tumor tissues (excised) and other sectioned major organs (kidney, liver, and lung) of the tumor-bearing mice were fixed in 4% paraformaldehyde for 48 h, followed by dehydration. Formalin-fixed major organs were further processed and embedded in paraffin wax. Embedded organs were sliced with a microtome (5 μm thick) and the sections were seared at 37 °C for 24 h. Hematoxylin and eosin Y staining was performed in tissue sections [12, 31–33]. Slides were processed and mounted with D.P.X. Different organ sections were observed under the microscope and used for image analysis.

### Immunohistochemistry studies of MDA-MB-231 xenografted mice tumor and other organs

Immunohistochemistry studies were performed according to the published protocol on 5-μM-thick tissue [31–33]. MDA-MB-231 cell-induced tumors were generated to demonstrate the GRP receptor expression. MDA-MB-231 tumor-bearing mice were sacrificed; tumors and other important organs (liver, lung, and kidney) were frozen in OCT embedding medium. Cryosections were cut into 5 μm and subjected to staining. Fixed tissue sections were rehydrated with graded alcohols and then rinsed with water. Quenching of endogenous peroxidase activity was done by incubating slides in 3% hydrogen peroxide in a light impermeable chamber. After PBS washing, slides were kept for 20 min in blocking solution [5% bovine serum albumin (*V*/*V*)]. The slides were then washed in PBS. Primary antibodies anti-GRPR (Abcam) were applied at 1:100 dilution and the tissues incubated for overnight in a humidity chamber. After washing again in PBS, the tissues were incubated for 2 h with horseradish peroxidase conjugated anti-rabbit IgG for GRPR. Incubating slides with Liquid DAB Substrate–Chromogen System (BD Pharmingen, CA, USA) for 2 min identified bound antibodies. After PBS and distilled water washing, the slides were counterstained with Mayer’s hematoxylin for the indicated period of time (usually one minute unless otherwise specified), dehydrated in graded alcohols, and mounted with a coverslip. Then the slides were counterstained with Mayer’s hematoxylin for the indicated period of time (usually 2 min), followed by a dehydration process using graded alcohols, and xylene. One drop of DPX permanent mounting medium was then immediately added to the tissue slices and was permanently mounted with a coverslip. All specimens were evaluated using an Olympus BX61 microscope with × 20 objectives. For negative controls, the primary antibody was omitted from the above protocol.

### Western blot analysis

Western blot analysis was performed by standard protocol [33–35], to study the intracellular protein expression of MDA-MB-231 cells, after treatment with the BNs. After washing in ice-cold PBS, proteins were extracted from cells with lysis buffer containing 100 mmol/L Tris-HCl, pH 8.0, 150 mmol/L NaCl, 1% sodium dodecyl sulfate, 10% glycerol, 1% Triton X, 5 mmol/L ethylenediaminetetraacetic acid, 1 mmol/L phenyl methyl sulfonyl fluoride, 10 mmol/L sodium fluoride, 1 mmol/L sodium orthovanadate, 1 mmol/l-glycerophosphate, and protease inhibitor cocktail tablets (Roche, Switzerland). Total proteins from cell were also extracted by using standard procedure [33, 34]. Protein concentrations were determined by Bradford reagent (BioRad, USA). Extracted proteins were separated by sodium dodecyl sulphate polyacrylamide gel electrophoresis and transferred to polyvinylidene fluoride (PVDF) membranes. Membranes blocking were done by 5% nonfat milk in Tris-buffered saline containing 0.1% polysorbate-20 and incubated with primary antibodies GRPR (Abcam, USA), VEGF, PCNA, Phospho-p38, and HIF-1α (Santa Cruz Biotechnology) for overnight at 4 °C. β-actin antibodies (Millipore) were used to confirm equal protein loading. Detection of primary antibodies was done by alkaline phosphatase conjugated secondary antibodies (1:5000 dilutions) and the detection was done by colorimetry using NBT-BCIP as substrate (Sigma-Aldrich). Detection of GRPR primary antibody was done by horseradish peroxidase conjugated secondary antibodies. The level of β-actin was used as a loading control and was not altered under any of the condition used.

### Angiogenic and proliferative markers in tumor

Tumor-bearing mice were intravenously treated with BN4 peptide at every day for 2 weeks as BN4 has shown the highest affinity and stability to GRP with compare to other analogs. Mice were sacrificed and tumor tissue sections were used for immunohistochemical (IHC) analyses. IHC was performed by the aforementioned methods [31, 33–36] with the primary antibodies such as anti-CD31 and anti-Ki67 (Abcam, Cambridge, Massachusetts, USA) to determine the expression of angiogenic and proliferative markers in the tumor.

### In vivo biodistribution studies

In tissue biodistribution studies, tumor-bearing animals were divided into four groups and well hydrated for 1 h by intraperitoneal administration of saline (0.9%, 2 mL). Three animals per groups were used for biodistribution studies. After that, the radiolabeled new bombesin analogs (^99m^Tc-BN) were injected intravenously in each animal of each group consequently through the tail vein [24–27]. After 5, 30, 60, and 120 min intravenous administrations of radiolabeled analogs, the animals were sacrificed; blood, heart, lung, liver, kidney, stomach, intestine, spleen, pancreas, brain, muscle, and tumor were excised, weighed, and counted for radioactivity. The organ uptake was calculated as a percentage of the injected activity per gram of wet tissue (%IA/g). In order to evaluate whether binding of bombesin analogs to MDA-MB-231 cells in vivo is specific; blocking studies with ^99m^Tc-labeled bombesin analogs in tumor-bearing mice were carried out. Groups of three animals were injected 0.03 nmol of ^99m^Tc-labeled BN analogs (2 MBq) with 25 nmol of non-radiolabeled BN peptide. After 60 min and 120 min, mice were sacrificed and the organs were collected. Each organ was weighed and counted in a gamma counter along with activity standard and percentage of injected activity per gram (%IA/g) of tissue was calculated for each type of tissues. Counts were corrected for background radiation and physical decay of the radioactivity. The uptake was expressed in the percentage of injected activity per gram of each organ (%IA/g). Data were analyzed statistically by the *t* test, with the level of significance set at *P* < 0.05.

### Imaging studies

In order to further demonstrate the biological performance of ^99m^Tc-BN1/^99m^Tc-BN2/^99m^Tc-BN3/^99m^Tc-BN4/^99m^Tc-BNS complexes, the imaging studies of radiotracers in MDA-MB-231 tumor-bearing female mice under anesthesia were performed. Three animals per groups were used for imaging studies. The imaging studies were performed on the female BALB/c mice bearing the MDA-MB-231 tumors at Thakurpukur Cancer Research Centre (Regional Radiation Monitoring Centre, Kolkata, India) under dual-head gamma camera (GE Hawkeyes, Pittsburgh, USA). Each tumor-bearing mouse was administered intravenously with radiolabeled analogs (22.2 MBq/mice) in 0.1 mL saline and was placed in a typical position for planar imaging under a small field of view experimental gamma camera [4, 25–27, 37], suitable for both planar and tomographic imaging. For blocking test, 200 μg free bombesin was intravenously injected into MDA-MB-231 tumor-bearing mice 30 min before ^99m^Tc-BN3 was injected. Static images were then acquired at 30 min and 120 min post-injection time using a GE Infinia gamma camera equipped with Xeleris Workstation. All the images taken were anterior images and analyzed using a gamma camera (GE Hawkeyes) fitted with a low-energy high-resolution all-purpose collimator using the static procedure of the Xeleris (Functional Imaging) Workstation system. After completion of imaging, animals were sacrificed by cervical dislocation.

### Statistical analysis

All experiments were repeated for at least three times. Results were expressed as mean and standard deviation (± SD). Statistical analysis has analyzed by one-way ANOVA. Graphs were prepared using ORIGIN 8.0 and GraphPad Prism Software. *P* values < 0.05 were considered statistically significant and calculations were done using Origin software.

## Results

### Synthesis of bombesin peptide analogs

**Scheme 1:**
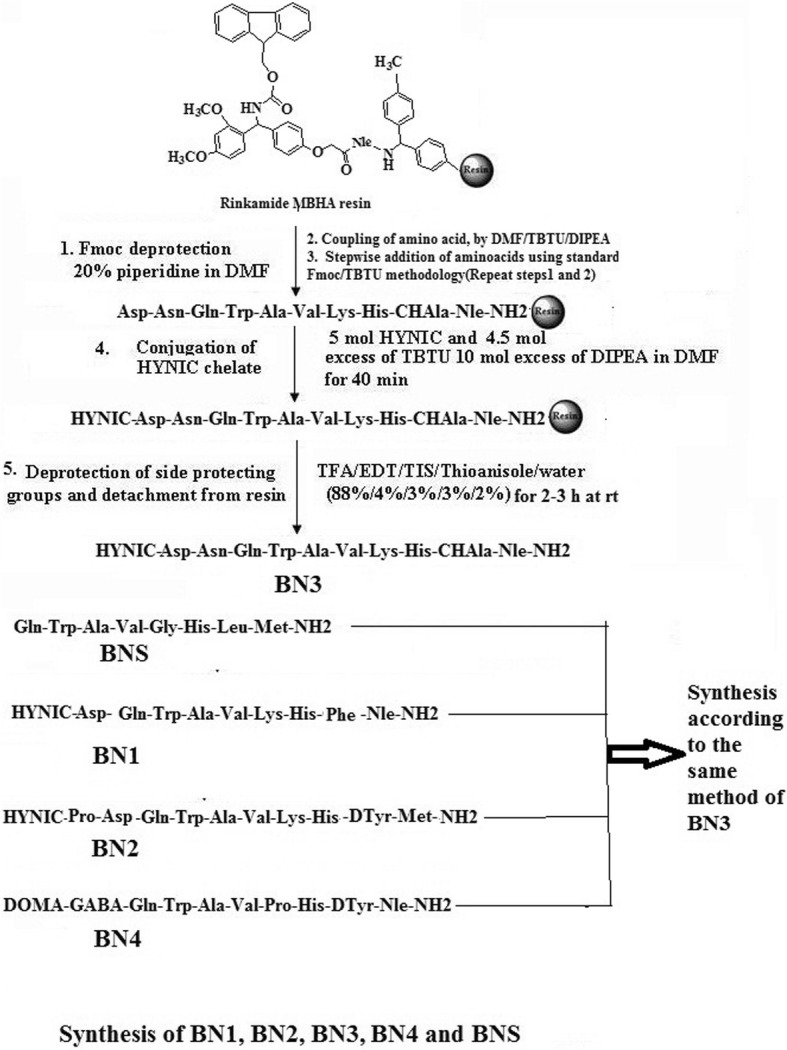
Solid-phase peptide synthesis of BN1, BN2, BN3, BN4, and BNS

**Fig. 1:**
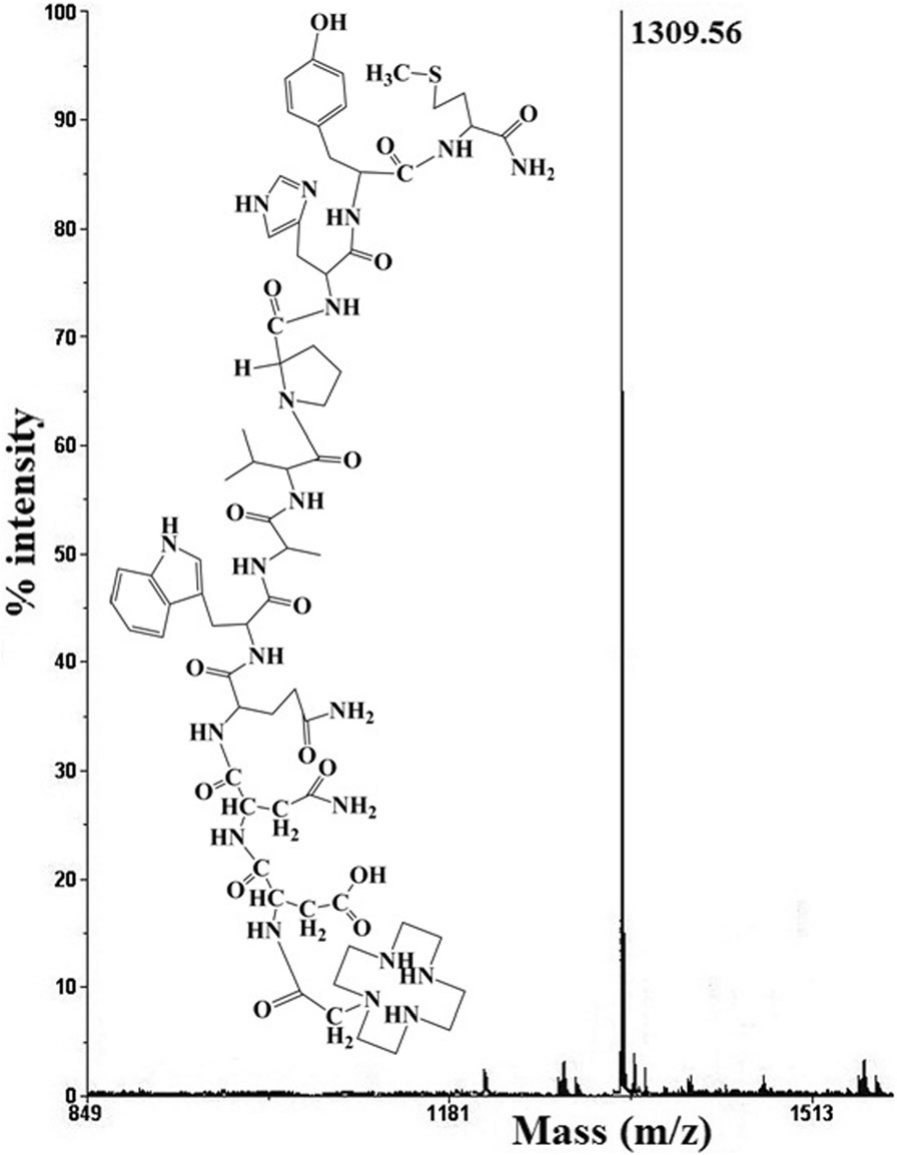
Chemical structure and MALDI mass spectra of new bombesin peptide DOMA-GABA-[Pro^5^-d-Tyr^13^Nle^14^]BN(7-14)NH_2_ (BN4)

**Fig. 2:**
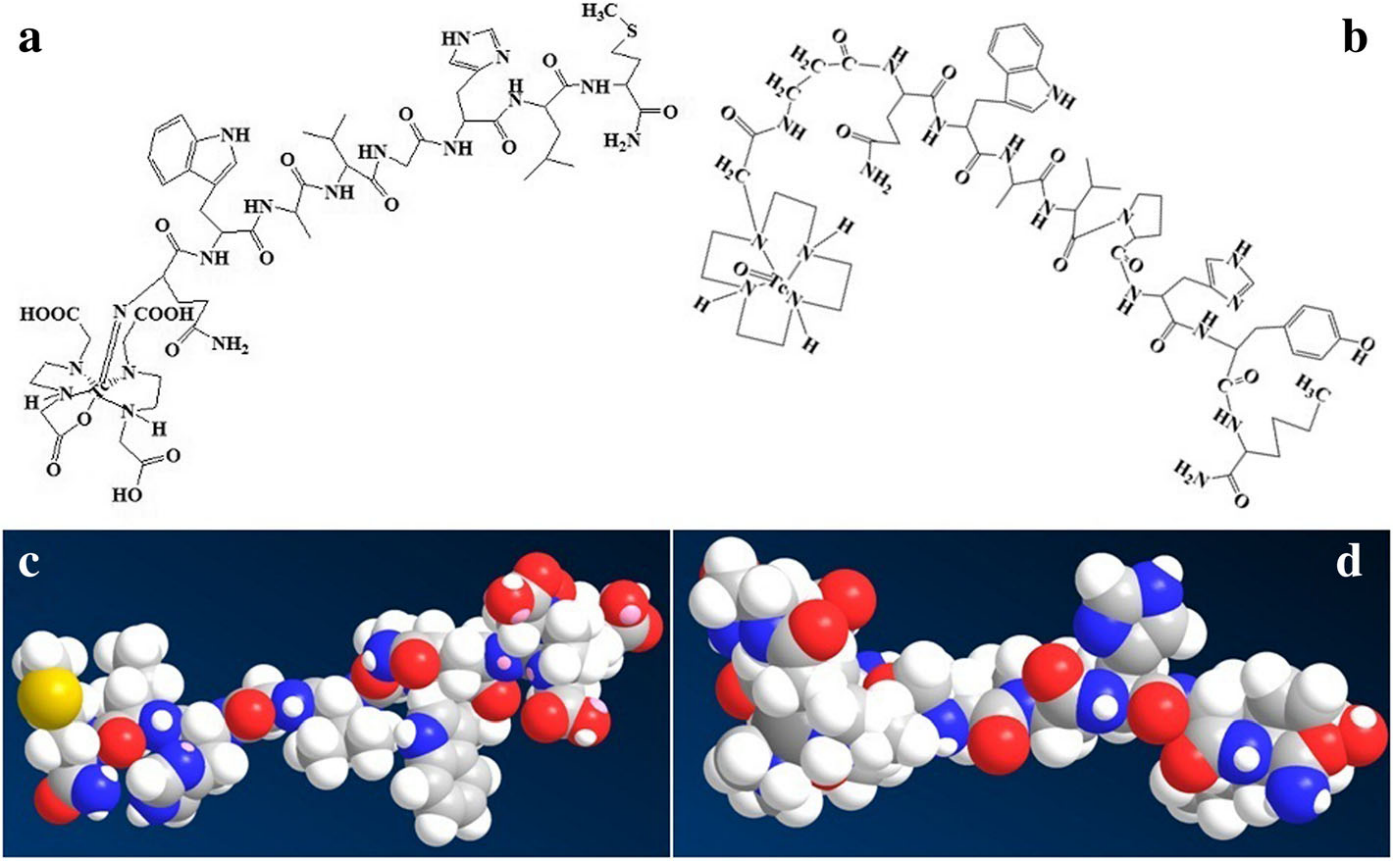
Chemical structure of ^99m^Tc-labeled BNS (**a**) and ^99m^Tc-labeled BN4 (**b**) with the MM2 energy minimized probable molecular structure of ^99m^Tc-BNS (**c**) and ^99m^Tc-BN4 (**d**)

All the bombesin peptide analogs were synthesized by Fmoc-based solid-phase peptide synthesis using Rink amide MBHA resin and the TBTU/*N*,*N*-diisopropylethylamine (DIPEA) coupling strategy (Scheme 1, supplementary section). After purification by HPLC, the overall yields of the conjugates were found to be in the range of 50% to 59% based on the removal of the first Fmoc group. More than 97% purity could be achieved after HPLC purification for all the analogs. All the purified peptides (white powder) were characterized by MALDI-TOF mass spectrometry, as shown in Fig. 1 for the BN4 analog, along with the proposed structure has been shown as a representative. Reports on the other synthesized peptides are shown in Additional file 1: Figure S1. The calculated molecular weight *m*/*z* (M + H)^+^ of BN1, BN2, BN3, BN4, and BNS were 1205.57, 1336.58, 1396.74, 1308.75, and 939.47 Da respectively, whereas the experimentally observed molar masses were 1206.31, 1337.46, 1397.58, 1309.56, and 940.12 Da for BN1, BN2, BN3, BN4, and BNS respectively. HPLC retention time was 19.35 min for BN1, 18.65 min for BN2, 17.31 min for BN3, 17.83 min for BN4, and 21.80 for BNS respectively. Results suggest that HYNIC and DOMA ligands could be successfully coupled to bombesin analogs (Fig. 2).

### Radiolabeling experiments

**Fig. 3:**
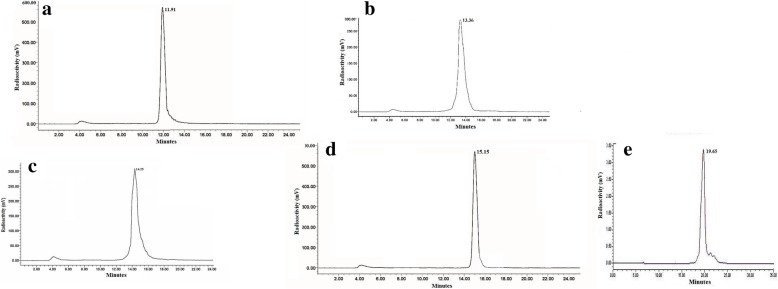
HPLC profiles of 99mTc-labeled BN analogs [**a**: for ^99m^Tc-BN1; **b**: for ^99m^Tc-BN2; **c**: for ^99m^Tc-BN3; **d** : for ^99m^Tc-BN4; **e**: for ^99m^Tc-BNS] used in this study to confirm the radiochemical purities

Radiolabeled conjugates were obtained by incubation of HYNIC/DOMA conjugated peptide analogs with ^99m^TCO_4_^−^ in the presence of excess tricine/EDDA and SnCl_2_. More than 95% radiochemical purity could be achieved after analytical radio HPLC purification. HPLC profiles of ^99m^Tc-labeled BN analogs used in this study have been shown in Fig. 3 to confirm the radiochemical purities. ITLC chromatograms were devoid of any significant change in radiochemical purity during the time course considered for the analysis with the values > 95%. Relatively higher radiochemical yield (95.58 ± 0.2%) with very low amount of free ^99m^Tc-pertechnetate (1.5 ± 0.02%), ^99m^Tc-radiocolloid (1.22 ± 0.08%), and ^99m^Tc-coligand (1.7 ± 0.02%) were recorded. RP-HPLC analysis of synthesized ^99m^Tc-labeled BN analogs consecutively showed bulk radioactivity eluting as a single major peak (*R*_t_ = 11.91, 13.36, 14.25, and 15.15 min respectively) and for ^99m^Tc-BNS major peak appeared at 19.65 min (*R*_t_), which was stable up to 24 h post-labeling period. In case of ITLC using 2-butanone as the solvent, the labeled ^99m^Tc-BNs, ^99m^Tc-coligands, and ^99m^Tc-colloid remain at the origin (*R*_*f*_ = 0) and ^99m^Tc-pertechnetate moved at the solvent front (*R*_*f*_ = 1). For 0.1 M sodium citrate at pH 5.1, the labeled ^99m^Tc-BNs and ^99m^Tc-colloid remained at origin (*R*_*f*_ = 0), ^99m^Tc-coligands and ^99m^Tc-pertechnetate moved at the solvent front (*R*_*f*_ = 1). On the other hand, for methanol/1 M ammonium acetate (1:1), ^99m^Tc-colloid remained at origin (*R*_*f*_ = 0) and labeled ^99m^Tc-BNs, ^99m^Tc-coligands, and ^99m^Tc-pertechnetate moved at the solvent front ((*R*_*f*_ = 1). The probable structures of ^99m^Tc-BNS and ^99m^Tc-BN4 are shown in Fig. 2 and similar information on the rest analogs are shown in Additional file 1: Figure S2.

The expected structure of these radiochemical complexes was correlated from previous studies (18–20) and the MM2 energy minimized structures by energy minimization computation of all possible structure using Chem-3D ultra software (Fig. 2c, d and Additional file 1: Figure S2). ^99m^Tc-BNS was purified by RP-HPLC and ITLC method like new radiolabeled peptide analogs and was used as a standard.

### Metabolic stability

In vitro stability of ^99m^Tc-labeled bombesin analogs incubated at 37 °C with either freshly collected rat serum or normal saline were monitored up to 24 h. The new radiolabeled peptides exhibited high in vitro stability both in phosphate buffer saline (pH = 7.4) and serum. The new radiolabeled peptide conjugates were stable up to 24 h post labeling period at room temperature, with no observable degradation or transchelation to serum proteins. Results for the more significant analogs ^99m^Tc-BN4, ^99m^Tc-BN3, and ^99m^Tc-BNS (standard analog), as shown in Table 1, suggest that the complexes are substantially stable for further in vitro/in vivo studies.

**Table 1:** In vitro stability of ^99m^Tc-BN3, ^99m^Tc-BN4, and ^99m^Tc-BNS in PBS and FBS after incubation for 2 to 24 h at 37 °C. Data were expressed as the mean ± SD

Time	% of radiolabeled peptides remaining					
	In FBS			In PBS		
	^99m^Tc-BN4	^99m^Tc-BN3	^99m^Tc-BNS	^99m^Tc-BN4	^99m^Tc-BN3	^99m^Tc-BNS
2 h	98.30 ± 0.10	97.50 ± 0.05	96.00 ± 0.04	95.50 ± 0.03	93.21 ± 0.20	94.31 ± 0.04
4 h	97.60 ± 0.05	97.00 ± 0.06	95.50 ± 0.07	95.00 ± 0.07	90.13 ± 0.05	90.22 ± 0.21
6 h	97.00 ± 0.18	96.50 ± 0.15	94.30 ± 0.12	93.50 ± 0.12	89.02 ± 0.07	86.10 ± 0.18
8 h	96.40 ± 0.20	95.00 ± 0.12	93.50 ± 0.18	92.50 ± 0.20	87.11 ± 0.12	82.14 ± 0.11
12 h	95.20 ± 0.05	94.00 ± 0.20	92.20 ± 0.05	90.00 ± 0.11	84.01 ± 0.21	80.10 ± 0.07
24 h	93.30 ± 0.02	90.00 ± 0.24	91.00 ± 0.07	88.00 ± 0.14	80.21 ± 0.16	77.17 ± 0.05

High labeling yield and stability was due to the optimization of the condition in judiciously choosing the amount of materials and also in the labeling method. All the new analogs, based on fragment BN(7-14) with the changes at positions 13, 14 (Cha and Nle for Leu and Met respectively) showed increased stability compared to the unmodified BNS. The instant radiolabeling yield of ^99m^Tc complexes of BN1, BN2, BN3, and BN4 were 95.44, 96.32, 96, and 97.12% respectively. The reduction in radiolabeling yield was insignificant (1–2%) after incubation for 24 h with saline. The level of free radioactive species generated after 24-h incubation in rat serum was ~ 8% in the case of radiolabeled BN1 and BN2, and about 5% for radiolabeled BN3 and BN4.

### Cysteine and histidine challenge

In vitro stability studies against ligand exchange and/or chemical decomposition, as determined through histidine/cysteine challenge, showed that the presence of His (strong competitor for ^99m^Tc) did not lead to remarkable instability. High affinity chelating system usually provides strong attachment of a radiometal to the ligand, thus minimizing the rate and amount of transchelation to other competing ligand such as cysteine and histidine, resulting in its extended shelf-life. For this reason, bifunctional chelators HYNIC/DOMA, known to form in vivo stable complex with ^99m^Tc, were used [24–26, 38, 39]. However, results showed that incubation of ^99m^Tc-labeled conjugates in the presence of excess cysteine/histidine (~ 100-fold molar excess) did not produce any significant transchelation to cysteine and histidine. A maximum of 10% radioactivity displacement by cysteine indicates the high bond strength of these compounds radiolabeled via HYNIC/DOMA. Results indicate high transchelation stability (88 to 95% at 6 h and from 85 to 90% at 24 h) of ^99m^Tc-labeled BN1, BN2, BN3, and BN4.

### Partition coefficient

The partition coefficient of the radiolabeled analogs was determined by distribution in phosphate buffer (pH 7.4) and *n*-octanol as described in the experimental section. *logD* values of the ^99m^Tc-BN1, ^99m^Tc-BN2, ^99m^Tc-BN3, ^99m^Tc-BN4, and ^99m^Tc-BNS were − 1.85 ± 0.05, − 1.78 ± 0.04, − 2.21 ± 0.13, –2.70 ± 0.06, and − 0.42 ± 0.10 respectively, reflecting their hydrophilic nature, due to the presence of additional amino acids in the peptide chains.

### Protein binding studies

Percentage of plasma protein binding for ^99m^Tc-BN1, ^99m^Tc-BN2, ^99m^Tc-BN3, and ^99m^Tc-BN4 in rat blood plasma was 45.00, 41.23, 26.00, and 19.10%, whereas the same for ^99m^Tc-BNS was 54%. The lower values of ^99m^Tc-BN3 and ^99m^Tc-BN4 indicate that a higher amount of the free radiotracer molecules are available for binding to the targeted receptor. Binding values mildly increased after 4 h for ^99m^Tc-BN1, ^99m^Tc-BN2, and ^99m^Tc-BNS, due to slow ligand exchange of the tricine for protein side chain. In general, the protein binding has a significant influence on the receptor uptake studies that need minimization in enhancing the receptor binding affinity of the radiotracer.

### Receptor binding affinity

**Fig. 4:**
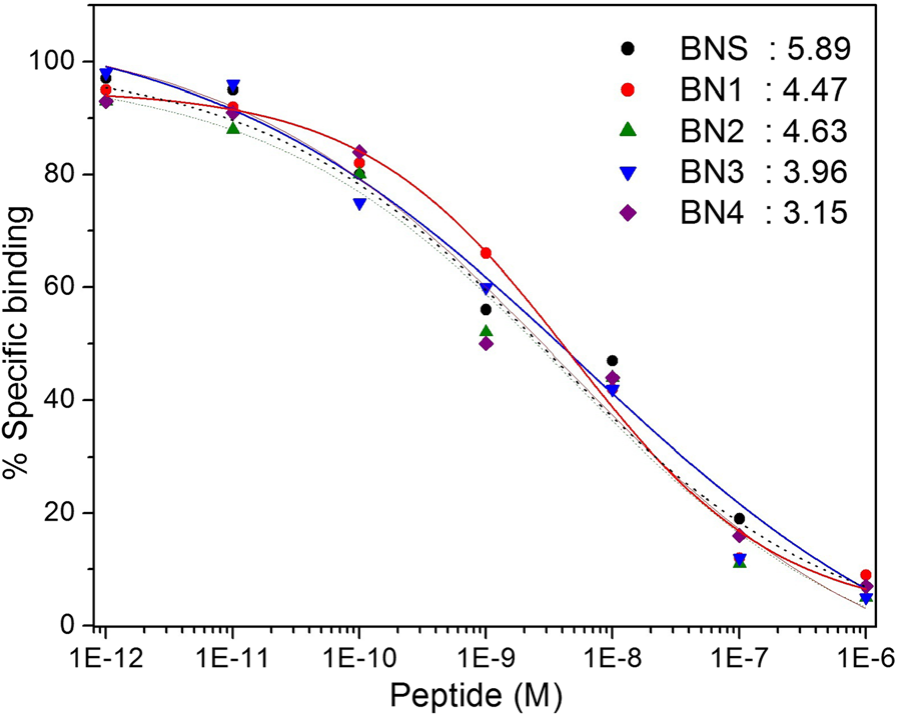
Receptor binding experiments of using GRP receptor expressing MDA-MB-231 cells. Peptide analogs, along with their IC50 values (in nM) are mentioned inside the figure

The affinity of the unlabeled analogs (BN1, BN2, BN3, and BN4 and BNS) toward GRPR was studied in human MDA-MB-231 cell membrane homogenates by inhibition experiments in competition with ^99m^Tc-BNS as radioligand [24–26, 38, 39], known to express an affinity for this receptor. Figure 4 shows the binding affinities of all the analogs to human cancer cell MDA-MB-231 overexpressing GRPR. The IC_50_ values were 3.96, 4.36, 3.15, 2.53 nmol/L for BN1, BN2, BN3, and BN4 respectively. The values were obtained by performing complete displacement experiments with the radioligand ^99m^Tc-BNS on membranes from cells expressing the GRP receptors and were compared with data for BNS. Displacement studies revealed that BN1, BN2, BN3, and BN4 when compared to BNS, inhibited the binding of ^99m^Tc-BNS in a monophasic manner. Competitive binding curves, summarized in Fig. 4, suggest that the BN1, BN2, BN3, and BN4 have higher affinities to GRPR than standard BNS. Peptides bearing an electronegative atom in the side chain of the amino acid residue in position 14 display increased IC_50_ values than BNS, whereas BN4 exhibits highest receptor binding affinity among all the peptides. It may be mentioned that non-radioactive Re complex can be used in place of its unlabeled ligand to determine the binding affinity after complexation reaction. This is being proposed to use the non-radioactive Re complex in place of its unlabeled ligand to determine the binding affinity after complexation in future studies.

### Internalization and externalization studies

**Fig. 5:**
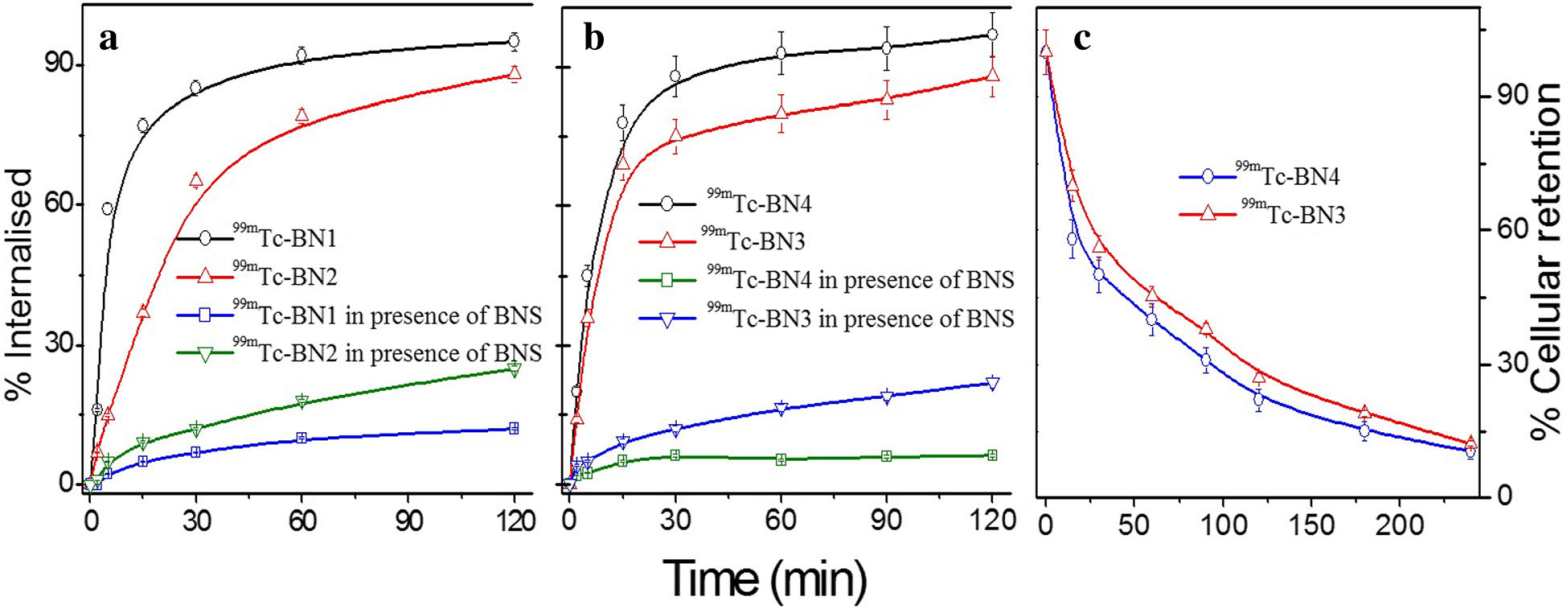
Internalization of ^99m^Tc-BN1 and ^99m^Tc-BN2 (**a**); ^99m^Tc-BN3 and ^99m^Tc-BN4 (**b**) into MDA-MB-231 cells at different time intervals. Nonspecific internalization is also presented by blocking experiment in the presence of excess BNS. Externalization of ^99m^Tc-BN3 and ^99m^Tc-BN4 (**c**) from MDA-MB-231 cells at different time intervals. Temperature at 37 °C

Receptor-specific time-dependent cellular internalization of radiolabeled compounds is expressed as the percent of added radioactivity per million cells. Receptor specificity of the cell binding and internalization of the radiolabeled conjugates were confirmed for all compounds by the addition of a 1000-fold excess of bombesin (referred to as blocking). Results showed similar and rapid uptake into MDA-MB-231 cells reaching a plateau between 30- and 120-min incubation periods at 37 °C. About 70 to 85% of the totally internalized ligand was specifically internalized in the cell lysate after stripping the surface bound activity of the cells in acidic buffer. The receptor blocking studies significantly decreased the internalization of radiolabeled compounds to < 0.5% in all cases in the presence of excess cold peptides. Within the first 2 h of incubation, ^99m^Tc-BN3 and ^99m^Tc-BN4 were internalized somewhat faster than ^99m^Tc-BN1 and ^99m^Tc-BN2; however, after 3 h, there was no statistical difference. All the internalization results are shown in Fig. 5a, b. After internalization, most of the ligand would be transported to the lysosomal compartment of the cells and then metabolized. The amount of the internalized radiopeptide was quantified using gamma ray spectrophotometer. The chemical forms of the radioactivity excreted from the cells were not estimated, although it is considered to be very important. Such a study is considered to be worth investigating to shed further light in this area of research.

The radiopeptides were allowed to internalize for a time period up to 120 min, the point at which internalization was considered to be maximum. After 240 min, the externalized radioactivity was measured. All the peptides showed significant fast efflux. Despite the high internalization rate, there was no long-term retention of radioactivity in the cells. The internalized radioactivity was released. After 30 min, 40 to 50% of the internalized radioconjugates got released. More than 55–70% was released after 180 min and about 78 to 90% was released after 24 h. The externalized peptides were intact that correspond to no indication of metabolites. The rate of externalization for ^99m^Tc-BN3 and ^99m^Tc-BN4 from cells are shown in Fig. 5c. Externalization curves of all the peptides reached a plateau after a certain time.

### Kinetics of blood clearance

**Fig. 6:**
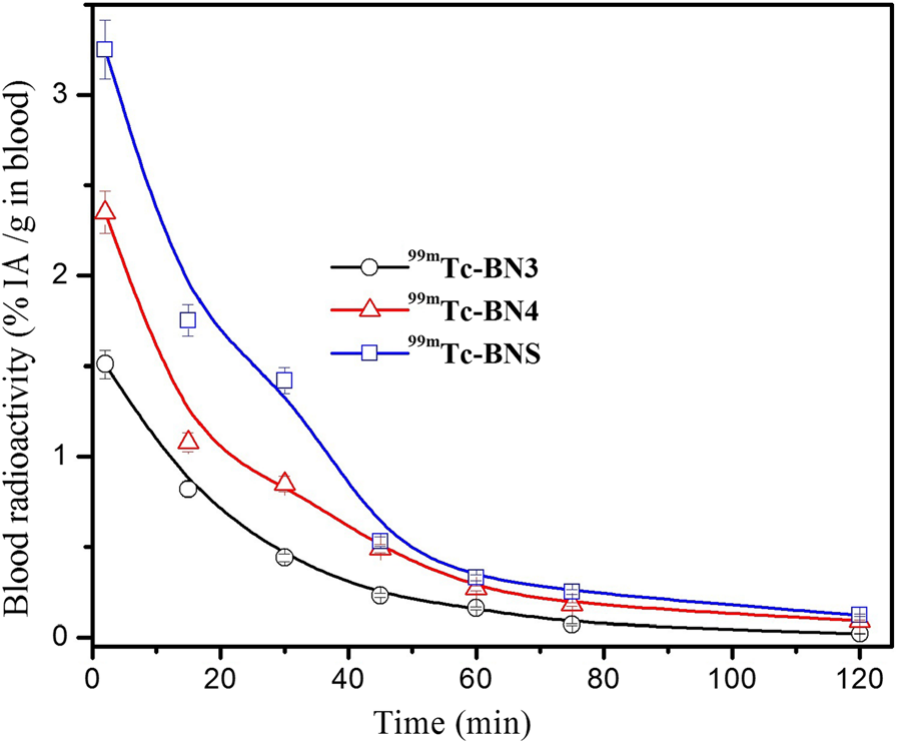
Blood clearance–time profile of radiolabeled peptides (mentioned inside the figure). An average of five experiments (*N* = 5) along with the mean ± SD have been presented

Blood clearance curves of ^99m^Tc-BN3, ^99m^Tc-BN4, and ^99m^Tc-BNS, as shown in Fig. 6, indicate that the radioactivity decreased to 0.02% at 120 min post-injection time with low retention in blood. *t*_1/2_ value of ^99m^Tc-BN4 in blood was 30 min. ^99m^Tc-labeled analogs showed rapid clearance of radioactivity from the blood circulation within 10, 15, and 20 min after which it gradually decreased (in the case of ^99m^Tc-BN3 and ^99m^Tc-BN4, clearance was very fast from the non-target tissues). The blood disappearance curves showed a biphasic pattern; an initial fast clearance wherein approximately 54–67% of activity was cleared within 10–25 min followed by a moderately slow decrease in which the rate of excretion was comparatively less.

### Tumor model formation, histological and immunohistochemical studies

**Fig. 7:**
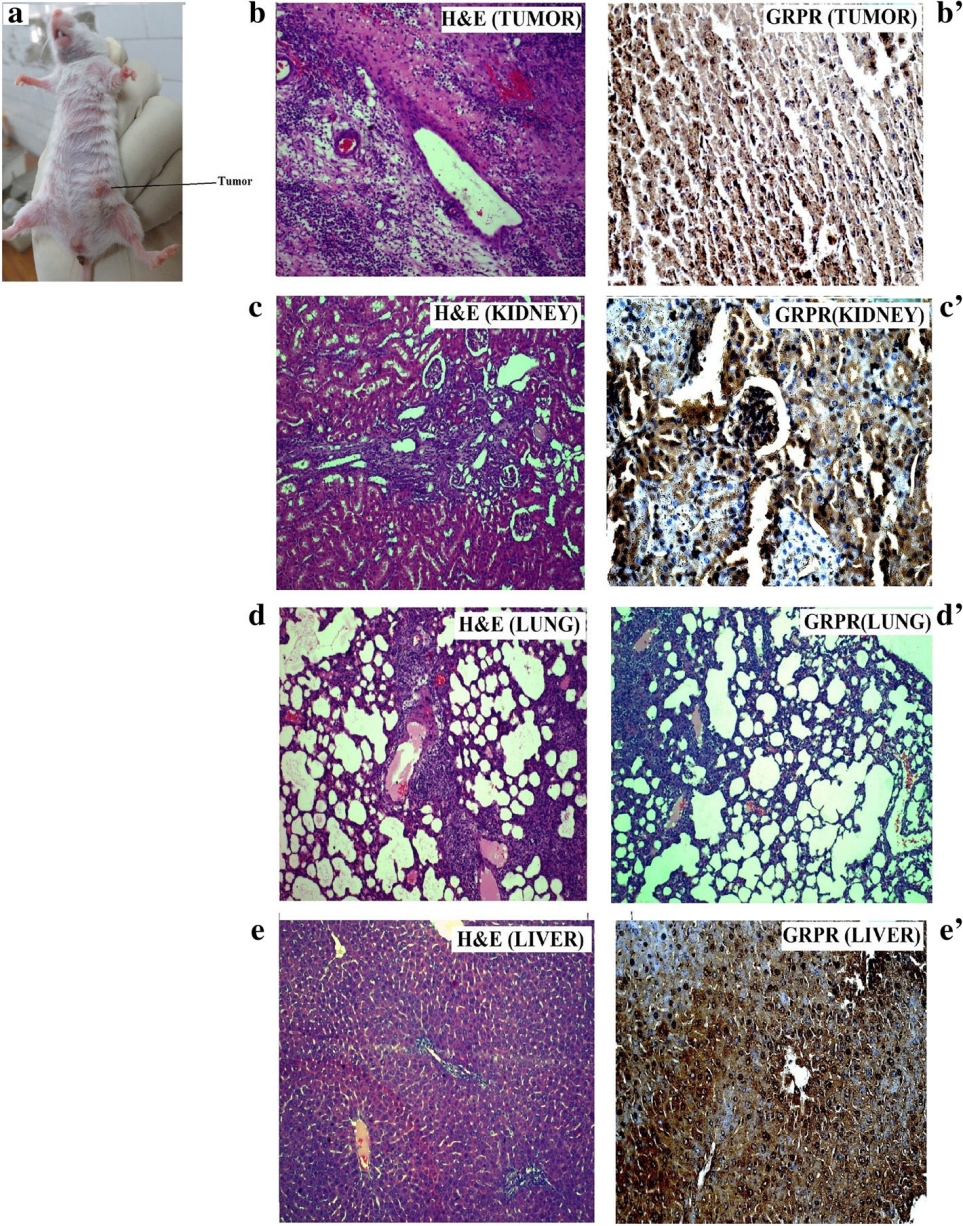
Histological and immunohistochemical micrographs of MDA-MB-231 cells induced mice tumor. (**a**) H&E staining of mice tumor section (**b**) and kidney section, (**c**) lung section, (**d**) liver section, and (**e**) excised from tumor-bearing mice and IHC staining for GRPR expression in mice tumor section (**b**′) and kidney section (**c**′), lung section (**d**′), and liver section (**e**′) of metastases tumor-bearing mice

Subcutaneous inoculation of MDA-MB-231 cells in the flank of left hind leg of the each mouse, tumorigenesis, and growth was found at the injection site (Fig. 7a). After cell implantation, the growth rate of tumor followed a satisfactory pattern of spheroid shape within 3 to 4 weeks (Fig. 7a). To eliminate the handling variations, tissue sections were prepared from the tumor so that they carry the adjacent portion of normal tissues. Histological evaluation of the tumor tissue section showed huge cell mass in fibrillary fashion, numerous mitoses, and nuclear atypia in tumor portion, whereas these features were totally absent in normal tissue portion (Fig. 7b). Histological analyses on important tissue sections (kidney, lung, liver) of tumor-bearing mice revealed that the sections presented (Fig. 7c, d) delicate conjunctive stroma and more necrosis in the central area and the presence of papillary projections and granular cells.

IHC studies (Fig. 7b′–e′) also reveal that GRPR protein is highly expressed in the mice tumor sections and also show macro metastases in the mice liver, lung, and kidney tissue sections. Both the results confirm the GRPR-specific mice breast tumor formation by MDA-MB-231 cells.

### Western blot analysis

**Fig. 8:**
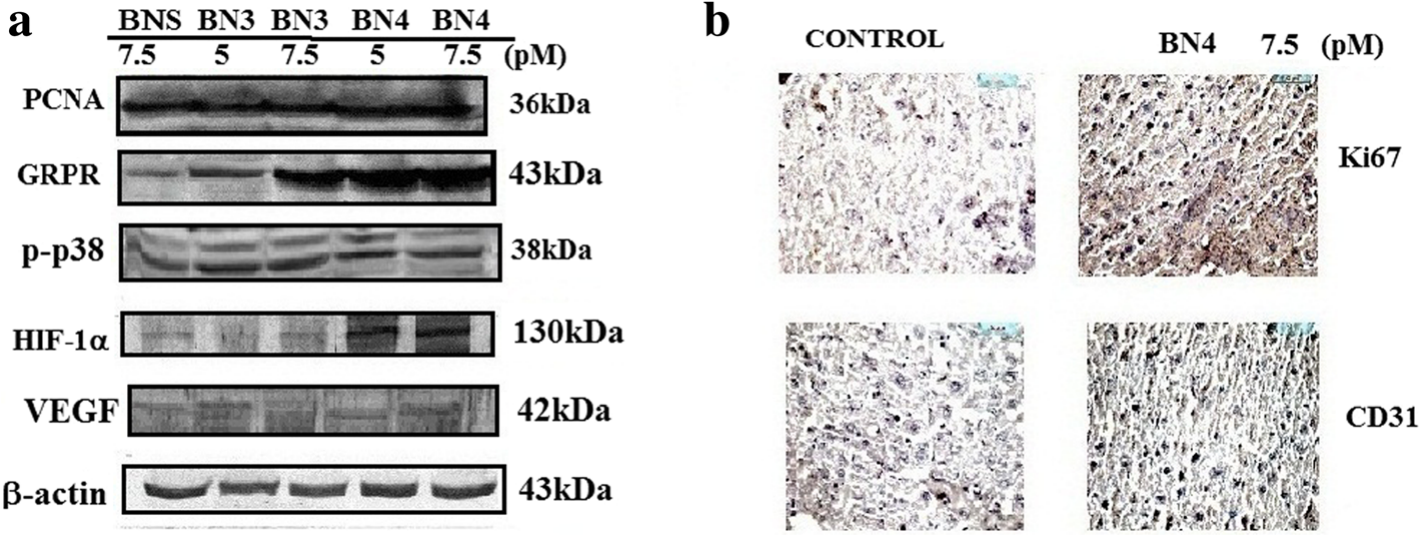
Western blot analyses of different protein expression such as PCNA, GRPR, p-p38, HIF-1α, p-53 in MDA-MB-231 cells treated with BNS, BN3, and BN4 at the indicated concentration. β-actin was used as the loading control. **a** Photomicrographs of immunohistochemical analyses of tumor sections, for expression of Ki-67 (cell proliferation marker) and CD-31 (angiogenesis marker) after BN4 treatment on tumor-bearing mice. **b** Results are representative of three independent experiments. The level of protein expression was found to increase gradually in a dose-dependent manner

From the H&E and IHC, elevated levels of GRPR in the mice tumor tissue section were found (Fig. 8). Moreover, these Western blot results also indicate about the confirmation of tumor formation by MDA-MB-231 cells. The activity of BN3/BN4/BNS on angiogenic proteins like HIF-1α and VEGF and at the same time GRPR proteins in MDA-MB-231 cells was examined. Here, all the data represent the treatment of MDA-MB-231 cells with BN3, BN4 (7.5 and 5 pM), and BNS at 7.5 pM concentration for 24 h showed the significantly increased level of GRPR (Fig. 8a) and dose-dependent elevation of GRPR. The Western blot analysis also showed an amplified level of HIF-1α and VEGF in cells. Activation of angiogenic signaling pathways is involved in HIF-1α, VEGF, and PCNA levels upregulation followed by BN3/BN4/BNS treatment. In parallel experiments, the levels of phosphorylation of p38 protein expressions followed by BN analog treatment were also checked by Western blot analysis. Dose-dependent increase in p38 protein contents was confirmed from the results. GRPR and other protein expressions distinctly increased with BN4 peptide treatment compared to BN3 and BNS; more metastasis and tumor growth was promoted via HIF-1α which increased angiogenesis. All the combined results suggest that BN4 exhibits a maximum effect on the cellular protein expression than BN3 and BNS as it has the highest binding affinity to GRPR among all the synthesized new bombesin analogs.

### Effect of bombesin analog on the expression of angiogenic and proliferative markers in tumor

In order to determine the effect of BN4 analog (that has the highest tumor targeting capability) on angiogenesis/proliferation in vivo, MDA-MB-231 tumor sections for cellular marker CD31 and Ki67 proteins were analyzed, an important signaling protein involved in angiogenesis, by IHC studies. IHC studies with anti-CD31 and anti-Ki67 antibodies in BN4 and BNS-treated MDA-MB-231 tumor sections are summarized in panel b of Fig. 8. BN4-treated tumor sections showed enhanced expression of CD31, Ki67, and appeared as dark brown staining in blood vessels and adjacent tissues. IHC studies also showed an increase in CD31 and Ki67 positively (Fig. 8b) at tumor tissues in BN4-treated animals compared with tumor tissues treated with BNS, suggesting that BN4 can stimulate angiogenesis and proliferation in MDA-MB-231 cell-induced tumor.

### Biodistribution study

**Fig. 9:**
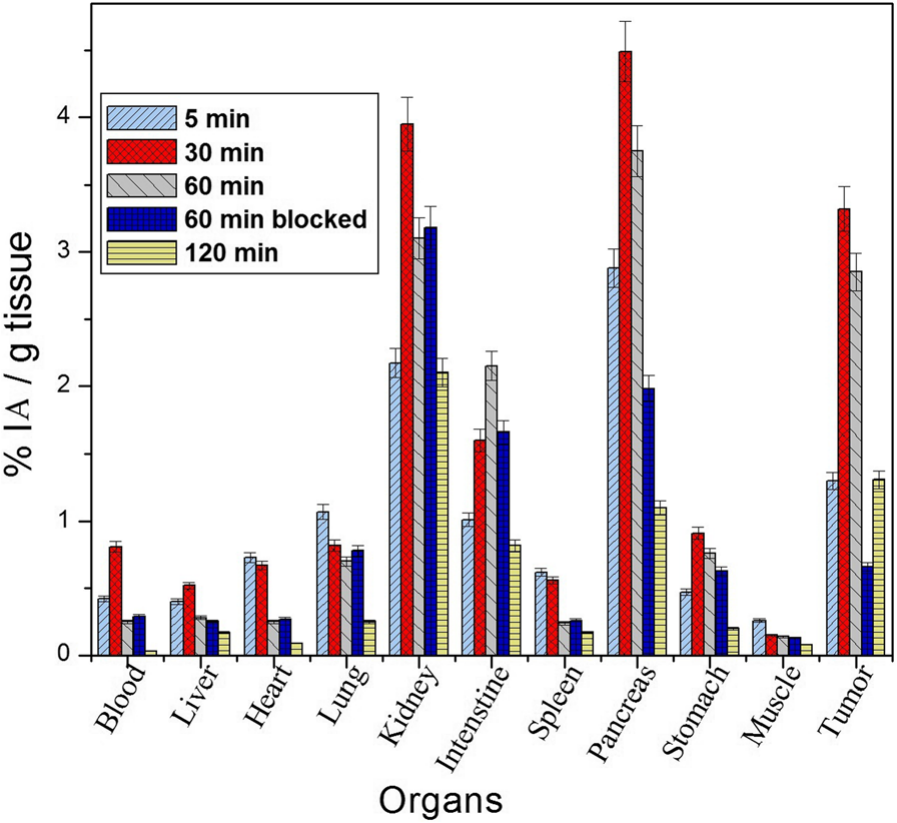
Biodistribution study of ^99m^Tc-BN4 in MDA-MB-231 cell-induced tumor-bearing mice at 5, 30, 60, and 120 min post injection with or without a co-administration of blocking peptide (BNS). All the data represent %IA/g values and each values are the mean ± SD of three animals per time point (*N* = 3)

Time-dependent biodistribution studies with the new radiotracers and ^99m^Tc-BNS were performed in mice-bearing MDA-MB-231 tumor xenografts. Biological distribution studies were made after intravenous administration of the ^99m^Tc-BN analogs, separately in MDA-MB-231 breast tumor-bearing mice which were sacrificed subsequently after 5, 30, 60, and 120 min, as summarized in Table 2 (for ^99m^Tc-BN1/^99m^Tc-BN2/^99m^Tc-BN3/^99m^Tc-BN4/^99m^Tc-BNS) and Fig. 9 (for ^99m^Tc-BN4, as representative). The results are expressed as the percentage of the injected activity per gram of the organ (%IA/g). After administration of ^99m^Tc-BN1/^99m^Tc-BN2/^99m^Tc-BN3/^99m^Tc-BN4, and ^99m^Tc-BNS, maximum uptake of the radioactivity was recorded in the kidney and exhibited higher blood levels even at 5 min p.i. (1.32 ± 0.07, 1.03 ± 0.05, 0.54 ± 0.01, 0.42 ± 0.01, and 1.25 ± 0.05 respectively). However, clearance from the blood pool was fast and efficient from the body of mice into the urine via the kidney and urinary tract as a result of its high hydrophilicity and providing low levels at 120 min p.i. for all the new analogs (≤ 0.05). Among the four new analogs, ^99m^Tc-BN3 and ^99m^Tc-BN4 showed relatively lower liver uptake at 30 p.i. (0.23 ± 0.13 and 0.32 ± 0.13) than ^99m^Tc-BN1, ^99m^Tc-BN2, and ^99m^Tc-BNS (0.69 ± 0.03,1.23 ± 0.06, and 0.85 ± 0.06 respectively). Radioactivity clearance from muscle and non-target tissues in case of ^99m^Tc-BN3 and ^99m^Tc-BN4 was higher; slightly slower washout for ^99m^Tc-BN1, ^99m^Tc-BN2, and ^99m^Tc-BNS, mainly via the kidney and the urinary system. Results also showed that significant uptake occurred in the GRPR-positive organs, including pancreas, intestine, and tumor; maximal tumor uptake was found at 30 min for all the compounds. ^99m^Tc-BN4 featured the highest accumulation at all p.i. times (1.30 ± 0.01, 3.32 ± 0.01, 2.85 ± 0.02, 1.31 ± 0.03 respectively) which was significantly higher than that of ^99m^Tc-BNS (0.45 ± 0.12, 0.95 ± 0.05, 0.75 ± 0.04, 0.58 ± 0.01). Higher uptake in the kidney than any other organs that gradually decreased as time passed for all four new analogs was noticed. The kidney uptake (%IA/g) of ^99m^Tc-BN4 was 2.17 ± 0.11, 3.95 ± 0.21, 3.10 ± 0.17, and 2.10 ± 0.13 at 5, 30, 60, and 120 min post administration. Almost a similar trend such as 2.86 ± 0.10, 3.12 ± 0.20, 2.82 ± 0.16, and 2.40 ± 0.16 at 5, 30, 60, and 120 min post administration was observed for ^99m^Tc-BNS in the biodistribution studies in breast tumor-bearing mice. Radioactivity was rapidly cleared from muscle and other non-target tissues. Relatively lower uptake of ^99m^Tc-BN4 in the stomach (0.47 ± 0.02, 0.51 ± 0.04, 0.36 ± 0.02, 0.12 ± 0.01) at all the time intervals (5, 30, 60, and 120 min) indicate minimal in vivo decomposition of the chelate to form free ^99m^TcO_4_/^99m^TcO_2_. As a whole, ^99m^Tc-BN4 and ^99m^Tc-BN3 were cleared more efficiently (*p* < 0.05) from background tissues and kidneys than ^99m^Tc-BN1, ^99m^Tc-BN2, and ^99m^Tc-BNS.

**Table 2:** Biodistribution data of the ^99m^Tc-labeled standard bombesin (BNS) and new bombesin analogs (BN1, BN2, BN3, BN4) in tumor-bearing mice at 5, 30, 60, and 120 min post-injection (PI). All the data represent %IA/g (mean ± SD) value and are the mean of results from three animals per time point

^99m^Tc-labeled BN analog	Organs	5 min	30 min	60 min	60 min (blocked)	120 min
(^99m^Tc-BNS)	Blood	1.25 ± 0.05	1.02 ± 0.03	0.82 ± 0.01	0.84 ± 0.01	0.54 ± 0.01
	Liver	1.60 ± 0.07	0.85 ± 0.06	0.40 ± 0.05	0.42 ± 0.04	0.22 ± 0.02
	Heart	1.80 ± 0.02	1.10 ± 0.01	0.79 ± 0.12	0.77 ± 0.11	0.50 ± 0.01
	Lung	1.35 ± 0.04	0.84 ± 0.11	0.68 ± 0.03	0.70 ± 0.05	0.33 ± 0.02
	Kidney	2.86 ± 0.10	3.12 ± 0.20	2.82 ± 0.16	2.88 ± 0.12	2.40 ± 0.12
	Intestine	0.84 ± 0.03	0.52 ± 0.05	0.31 ± 0.02	0.36 ± 0.02	0.22 ± 0.01
	Spleen	0.22 ± 0.04	0.18 ± 0.03	0.11 ± 0.02	0.10 ± 0.03	0.06 ± 0.11
	Pancreas	0.88 ± 0.01	1.73 ± 0.10	1.14 ± 0.05	0.90 ± 0.02	0.95 ± 0.04
	Stomach	0.76 ± 0.03	0.69 ± 0.05	0.46 ± 0.04	0.45 ± 0.01	0.12 ± 0.02
	Muscle	0.16 ± 0.03	0.26 ± 0.02	0.18 ± 0.04	0.17 ± 0.03	0.11 ± 0.02
	Tumor	0.45 ± 0.12	0.95 ± 0.05	0.75 ± 0.04	0.68 ± 0.20	0.58 ± 0.01
	Urine + urinary bladder	0.56 ± 0.02	1.92 ± 0.07	2.54 ± 0.22	2.58 ± 0.18	2.85 ± 0.03
(^99m^Tc-BN1)	Blood	1.32 ± 0.07	0.89 ± 0.04	0.32 ± 0.01	0.34 ± 0.01	0.05 ± 0.01
	Liver	0.85 ± 0.04	0.69 ± 0.03	0.56 ± 0.02	0.55 ± 0.03	0.32 ± 0.01
	Heart	1.02 ± 0.05	0.81 ± 0.04	0.47 ± 0.02	0.44 ± 0.01	0.17 ± 0.01
	Lung	0.95 ± 0.04	0.77 ± 0.03	0.56 ± 0.02	0.57 ± 0.02	0.32 ± 0.01
	Kidney	2.12 ± 0.10	4.12 ± 0.20	3.15 ± 0.15	3.10 ± 0.02	1.56 ± 0.07
	Intestine	1.06 ± 0.05	1.36 ± 0.06	1.70 ± 0.08	1.44 ± 0.05	1.10 ± 0.05
	Spleen	0.94 ± 0.04	0.51 ± 0.02	0.32 ± 0.01	0.35 ± 0.01	0.26 ± 0.03
	Pancreas	2.58 ± 0.12	1.79 ± 0.08	1.11 ± 0.05	0.91 ± 0.04	0.69 ± 0.03
	Stomach	0.42 ± 0.02	0.85 ± 0.04	0.56 ± 0.02	0.40 ± 0.02	0.35 ± 0.01
	Muscle	0.22 ± 0.02	0.27 ± 0.01	0.14 ± 0.01	0.16 ± 0.02	0.09 ± 0.02
	Tumor	0.78 ± 0.03	2.51 ± 0.12	1.85 ± 0.09	1.20 ± 0.03	1.56 ± 0.07
	Urine + urinary bladder	0.81 ± 0.04	2.85 ± 0.01	3.50 ± 0.15	3.56 ± 0.02	4.79 ± 0.12
(^99m^Tc-BN2)	Blood	1.03 ± 0.05	0.78 ± 0.03	0.35 ± 0.01	0.38 ± 0.01	0.07 ± 0.01
	Liver	1.47 ± 0.07	1.23 ± 0.06	1.01 ± 0.05	1.09 ± 0.04	0.53 ± 0.02
	Heart	0.59 ± 0.02	0.46 ± 0.01	0.23 ± 0.12	0.27 ± 0.02	0.12 ± 0.01
	Lung	0.91 ± 0.04	0.89 ± 0.11	0.78 ± 0.03	0.73 ± 0.04	0.42 ± 0.02
	Kidney	2.05 ± 0.10	4.76 ± 0.20	3.34 ± 0.16	3.37 ± 0.10	2.45 ± 0.12
	Intestine	0.73 ± 0.03	1.05 ± 0.05	1.46 ± 0.02	1.25 ± 0.02	0.18 ± 0.01
	Spleen	0.95 ± 0.04	0.75 ± 0.03	0.48 ± 0.02	0.45 ± 0.03	0.24 ± 0.11
	Pancreas	0.95 ± 0.01	2.07 ± 0.10	1.08 ± 0.05	0.85 ± 0.02	0.89 ± 0.04
	Stomach	0.74 ± 0.03	1.01 ± 0.05	0.82 ± 0.04	0.70 ± 0.02	0.41 ± 0.02
	Muscle	0.24 ± 0.01	0.20 ± 0.02	0.18 ± 0.03	0.20 ± 0.02	0.12 ± 0.04
	Tumor	0.79 ± 0.12	1.85 ± 0.05	0.98 ± 0.04	0.78 ± 0.03	0.54 ± 0.02
	Urine + urinary bladder	0.56 ± 0.02	1.92 ± 0.07	4.54 ± 0.22	4.50 ± 0.08	5.55 ± 0.03
(^99m^Tc-BN3)	Blood	0.54 ± 0.01	0.85 ± 0.01	0.47 ± 0.04	0.30 ± 0.01	0.24 ± 0.01
	Liver	0.44 ± 0.03	0.23 ± 0.13	0.18 ± 0.12	0.20 ± 0.03	0.11 ± 0.03
	Heart	0.91 ± 0.03	0.77 ± 0.02	0.35 ± 0.02	0.29 ± 0.11	0.15 ± 0.04
	Lung	0.87 ± 0.14	0.62 ± 0.03	0.50 ± 0.02	0.52 ± 0.02	0.35 ± 0.11
	Kidney	1.97 ± 0.11	3.88 ± 0.21	3.17 ± 0.17	3.18 ± 0.20	1.40 ± 0.13
	Intestine	1.21 ± 0.06	1.46 ± 0.05	1.85 ± 0.21	1.36 ± 0.23	0.70 ± 0.05
	Spleen	0.54 ± 0.04	0.46 ± 0.01	0.30 ± 0.01	0.33 ± 0.03	0.12 ± 0.02
	Pancreas	2.78 ± 0.11	3.59 ± 0.03	2.11 ± 0.06	0.92 ± 0.02	1.22 ± 0.04
	Stomach	0.52 ± 0.02	0.74 ± 0.04	0.66 ± 0.02	0.37 ± 0.10	0.25 ± 0.01
	Muscle	0.50 ± 0.01	0.30 ± 0.02	0.26 ± 0.03	0.16 ± 0.21	0.19 ± 0.02
	Tumor	1.28 ± 0.01	2.95 ± 0.01	2.70 ± 0.02	0.54 ± 0.20	1.69 ± 0.03
	Urine + urinary bladder	0.61 ± 0.04	2.80 ± 0.01	3.75 ± 0.11	3.52 ± 0.07	3.91 ± 0.22
(^99m^Tc-BN4)	Blood	0.42 ± 0.01	0.81 ± 0.01	0.42 ± 0.03	0.29 ± 0.01	0.17 ± 0.00
	Liver	0.40 ± 0.03	0.52 ± 0.13	0.28 ± 0.12	0.25 ± 0.03	0.17 ± 0.03
	Heart	0.73 ± 0.03	0.67 ± 0.02	0.25 ± 0.02	0.27 ± 0.11	0.09 ± 0.04
	Lung	1.07 ± 0.14	0.82 ± 0.03	0.70 ± 0.02	0.78 ± 0.02	0.25 ± 0.11
	Kidney	2.17 ± 0.11	3.95 ± 0.21	3.10 ± 0.17	3.18 ± 0.20	2.10 ± 0.13
	Intestine	1.01 ± 0.06	1.60 ± 0.05	2.15 ± 0.21	1.66 ± 0.23	0.82 ± 0.05
	Spleen	0.62 ± 0.04	0.56 ± 0.01	0.24 ± 0.01	0.26 ± 0.03	0.17 ± 0.02
	Pancreas	2.88 ± 0.11	4.49 ± 0.03	3.75 ± 0.06	1.98 ± 0.02	1.10 ± 0.04
	Stomach	0.47 ± 0.02	0.91 ± 0.04	0.76 ± 0.02	0.63 ± 0.10	0.20 ± 0.01
	Muscle	0.26 ± 0.02	0.32 ± 0.04	0.25 ± 0.02	0.13 ± 0.10	0.10 ± 0.01
	Tumor	1.30 ± 0.01	3.32 ± 0.01	2.85 ± 0.02	0.66 ± 0.20	1.31 ± 0.03
	Urine + urinarybladder	0.47 ± 0.04	2.65 ± 0.01	3.92 ± 0.11	3.95 ± 0.07	4.31 ± 0.22

**Fig. 10:**
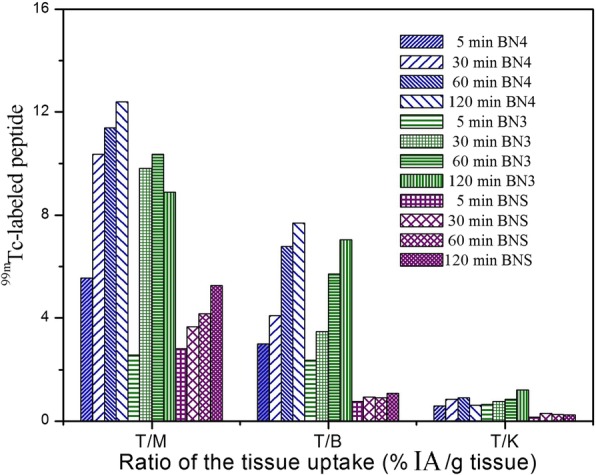
Comparative tumor to non-target tissue ratios of ^99m^Tc-BN3, ^99m^Tc-BN4, and ^99m^Tc-BNS in MDA-MB-231-induced tumor-bearing mice at 5, 30, 60, and 120 min post injection. T/M: tumor to muscle, T/B: tumor to blood, and T/K: tumor to kidney. All the data represent %IA/g values and each values are the mean ± SD of three animals per time point (*N* = 3)

GRP receptor-rich organs (pancreas, gastrointestinal tract, and breast tumor) exhibited higher uptake than other organs for all the new radiotracers (^99m^Tc-BN1, ^99m^Tc-BN2, ^99m^Tc-BN3, and ^99m^Tc-BN4). Among all the analogs, ^99m^Tc-BN4 and ^99m^Tc-BN3 displayed significantly (*p* < 0.05) higher kidney uptake than ^99m^Tc-BNS during the initial time intervals and ^99m^Tc-BNS showed slower washout from the kidney and background tissues over 2-h post injection. The organ uptakes were also found to be receptor specific, as a significant reduction (*p* < 0.01) of the uptake (Fig. 9, for ^99m^Tc-BN4) in the group of blocked animals (receiving 100 μg BNS). The reduction was > 80% in the breast tumor and > 75% in the case of the pancreas. No blocking was found in the liver, kidney, or spleen and was therefore considered to be the receptor-mediated process. The pancreas uptake of 3.59 ± 0.03, 4.49 ± 0.03% IA/g for ^99m^Tc-BN3 and ^99m^Tc-BN4 at 30 min post administration reached only 0.92 ± 0.02, 1.78 ± 0.02%IA/g in the group of blocked animals and breast tumor uptake 2.95 ± 0.01, 3.32 ± 0.01%IA/g at 30 min and significant (*p* < 0.01) reduction in tumor uptake (0.66 ± 0.20%IA/g) was observed in the blocked animals. No appreciable differences were observed for non-GRP receptor-positive organs between the mice that received a co-injection of blocking dose and unblocked mice. Initially, accumulation of ^99m^Tc-BN3 and ^99m^Tc-BN4 in breast tumors were not significantly different but at 30 min post injection, ^99m^Tc-BN4 uptake was significantly (*p* < 0.05) higher than ^99m^Tc-BN3. However, after 2-h post administration, kidney uptake values for ^99m^Tc-BNS were higher than ^99m^Tc-BN1, ^99m^Tc-BN2, ^99m^Tc-BN3, and ^99m^Tc-BN4. This is due to a greater degree of retention and slower wash out of ^99m^Tc-BNS from kidney and other background tissues compared to the new analogs at longer time of post injection. The higher tumor uptake and lower liver uptake with faster clearance from blood/normal tissues led to a high tumor-to-non tumor ratio. After 5, 30, 60, and 120 min p.i for the new analog, ^99m^Tc-BN3 tumor/kidney = 0.64, 0.76, 0.85, 1.20; tumor/muscle = 2.56, 9.83, 10.38, 8.89; and tumor/blood = 2.37, 3.47, 5.71, 7.04 respectively. In the case of ^99m^Tc-BN4 tumor/kidney = 0.59, 0.84, 0.91, 0.62; tumor/muscle = 5.00, 10.37, 11.40, 12.40; and tumor/blood = 3.00, 4.09, 6.78, 7.70 (Fig. 10).

Some important tumor-to-normal tissue uptake ratio has been shown in Fig. 10. While comparing the tumor-to-kidney ratio, ^99m^Tc-BN4 had the higher ratio (0.91) than the other analogs and ^99m^Tc-BNS (0.26 at 60 min post administration). As the background activity gradually got cleared from the body, there was an increase in the target to non-target ratio of ^99m^Tc-BN4 with the passage of time, such as tumor-to-blood ratio (4.09) and tumor-to-muscle ratio (10.37) were high while the tumor-to-kidney ratio (0.84) was low at 30 min post administration. The excretion results also demonstrate that ^99m^Tc-BN4 and ^99m^Tc-BN3 were cleared rapidly from the blood, heart, lung, and muscle into the urine via the kidney and urinary tract as a result of its higher hydrophilicity than ^99m^Tc-BN1, ^99m^Tc-BN2, and ^99m^Tc-BNS. Owing to the high tumor uptake and fast renal excretion, the accumulation of radioactivity tumor-to-muscle ratio were higher demonstrating no statistical difference between biodistribution and imaging studies.

### Imaging studies

**Fig. 11:**
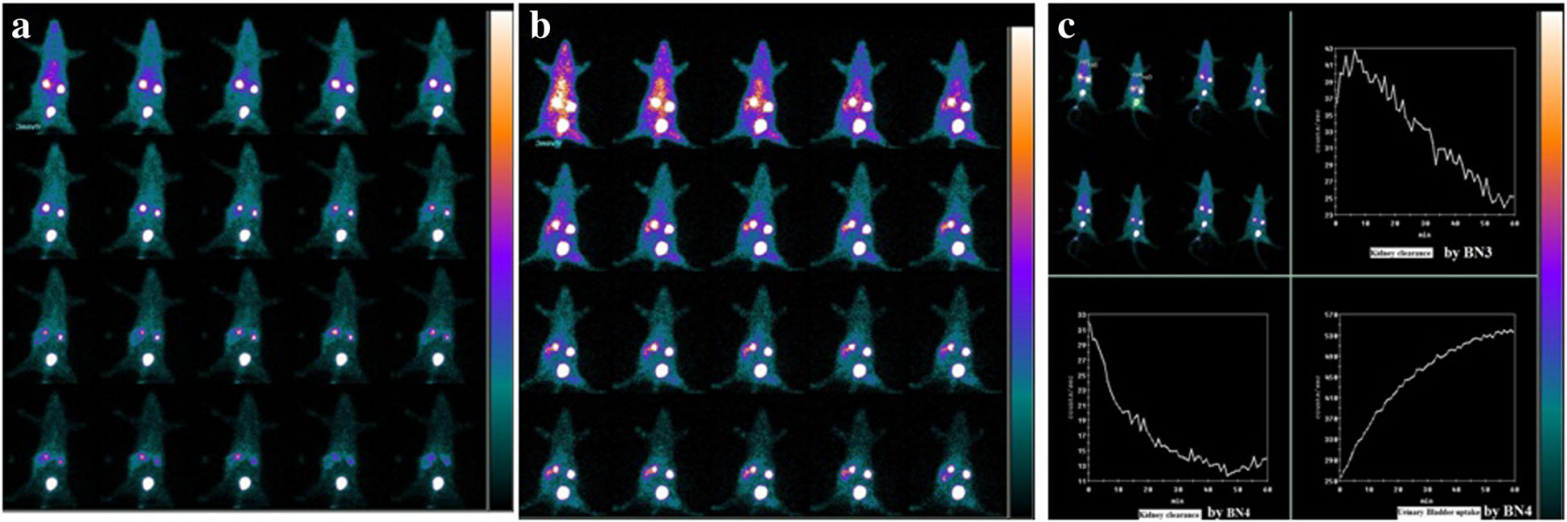
Scintigraphic images of MDA-MB-231 cell induced tumor bearing mice at different time period after intravenous injection of ^99m^Tc-BNS (**a**) and ^99m^Tc-BN4 (**b**). Gamma camera images with renogram curves (**c**) after intravenous administration of ^99m^Tc-BN3 and ^99m^Tc-BN4 in mice for 60 min. Curves suggest rapid urinary excretion, followed by gradual decline in the kidney uptake. Three animals were used for each group

Although the in vivo biodistribution studies can provide the quantitative assessment of organ uptake of the ^99m^Tc-labeled analogs, whole-body imaging studies allow direct comparison between the different organs for their imaging quality in living animals. To further demonstrate the biological performance of these new analogs and ^99m^Tc-BNS complexes, the imaging studies of all radiotracers in breast tumor-bearing mice under anesthesia were performed (three animals per groups were used for imaging studies). Dynamic planar images of ^99m^Tc-BNS on tumor-bearing mice have shown in Fig. 11a and for ^99m^Tc-BN4 on tumor-bearing mice have shown in Fig. 11b up to 60 min. The images have shown clear delineation of the tumor in the case of ^99m^Tc-BN4 and low abdominal uptake with urinary excretion.

**Fig. 12:**
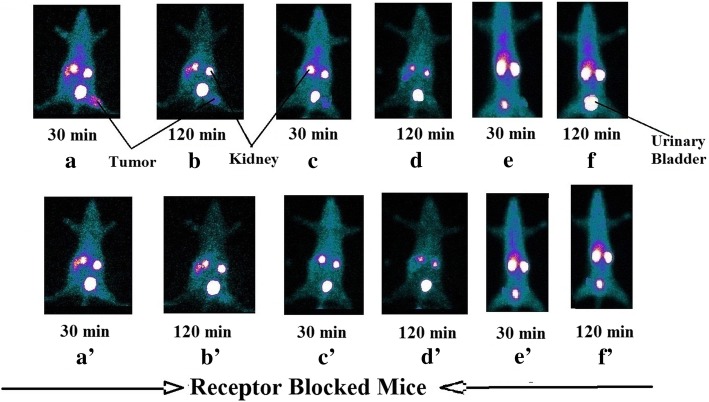
Planar static images of MDA-MB-231 tumor-bearing mice at different time period after i.v. injection of (**a**) ^99m^Tc-BN4 alone at 30 min, (**b**) ^99m^Tc-BN4 alone at 120 min, (**c**) ^99m^Tc-BN3 alone at 30 min, (**d**) ^99m^Tc-BN3 alone at 120 min, (**e**) ^99m^Tc-BNS alone at 30 min, and (**f**) ^99m^Tc-BNS alone at 120 min. (**a**′) ^99m^Tc-BN4 blocked by co-injection of BNS at 30 min. (**b**′) ^99m^Tc-BN4 blocked by co-injection of BNS at 120 min. (**c**′) ^99m^Tc-BN3 blocked by co-injection of BNS at 30 min. (**d**′) ^99m^Tc-BN3 blocked by co-injection of BNS at 120 min. (**e**′) ^99m^Tc-BNS blocked by co-injection of BNS at 30 min. (**f**′) ^99m^Tc-BNS blocked by co-injection of BNS at 120 min. Three animals were used for each group

Scintigraphic images can confirm the accumulation of the radiopharmaceutical in the tumor. In between all the analogs, ^99m^Tc-BN4 and ^99m^Tc-BN3 showed more considerable tumor uptake than ^99m^Tc-BN1, ^99m^Tc-BN2, and ^99m^Tc-BNS. Serial gamma camera images suggest urinary excretion, the main elimination pathway for all the analogs. Figure 11a, b have shown for ^99m^Tc-BN4, ^99m^Tc-BNS. Results also suggest higher uptake in the kidney than any other organs which gradually decreases as time passes and uptake in urinary bladder increases. This indicates that the kidney and urinary systems represent the predominant excretion route for all these radiopeptide analogs. Figure 11c represents images with renogram curves after intravenous administration of ^99m^Tc-BN4, ^99m^Tc-BN3 in mice for 60 min. Rapid urinary excretion, followed by a gradual decline in kidney uptake was noticed. Figure 12 represents the static images of MDA-MB-231-induced tumor-bearing mice at 30 and 120 min post-administration of ^99m^Tc-BN4, ^99m^Tc-BN3, and ^99m^Tc-BNS with and without a co-injected blocking dose of BNS. Higher uptake in the mice tumors was recorded at 30 min post-injection of ^99m^Tc-BN4. Conversely, the other group of mice that received blocking dose showed significantly reduced uptake at tumor sites (Fig. 12a′–f′). Prominent uptake was recorded in the kidneys among all the animals. Two hours after the administration of ^99m^Tc-BN4, ^99m^Tc-BN3 (Fig. 12b–d), tumor site in the non-blocked animals could still be identified, as well as higher uptake than other normal organs. Owing to the high tumor uptake and fast renal excretion, for the accumulation of radioactivity tumors to muscle ratios were high and low for the tumor-to-kidney ratios at 30 min post injection of ^99m^Tc-BN4, ^99m^Tc-BN3 demonstrating no statistical difference between biodistribution and scintigraphic studies. Tumors were clearly visualized at 30 min post injection with an excellent tumor to background contrast as a significant amount of radioactivity was associated with GRP-positive MDA-MB-231 tumor. Other prominent regions of ^99m^Tc-BN4, ^99m^Tc-BN3, and ^99m^Tc-BNS uptakes were the kidneys and bladder, due to rapid excretion of these compounds into the urine via the kidney. As shown in the static images, significant tumor uptake and excellent clearing properties of ^99m^Tc-BN4/^99m^Tc-BN3 from the background tissues and kidney than the others and ^99m^Tc-BNS are evident in the tumor-bearing animals at 30 min and 120 min post administration (Fig. 12). It may be mentioned that an image algebraic subtraction of the blocked animals from the non-blocked animals could provide further information regarding more specific binding studies. This is considered to be one of the future perspectives of the present work.

All these image studies are in concurrence with the biodistribution results of the ^99m^Tc-labeled BN analogs in tumor-bearing mice. However, ^99m^Tc-BN4/^99m^Tc-BN3 produce the most significant images comparatively fewer uptake images for ^99m^Tc-BN1, ^99m^Tc-BN2, and ^99m^Tc-BNS. As a result, the highest target-to-background ratios was exhibited by ^99m^Tc-BN4 followed by ^99m^Tc-BN3 illustrating the favorable characteristics of these agents for tumor imaging. Among all the analogs, during scintigraphic studies, tumor region was most clearly visualized in the case of ^99m^Tc-BN4 than ^99m^Tc-BN1, ^99m^Tc-BN2, ^99m^Tc-BN3, and ^99m^Tc-BNS. All these properties clearly proved that ^99m^Tc-labeled new analogs have GRP receptor expressing tumor visualization properties and comparing all results ^99m^Tc-BN4 and ^99m^Tc-BN3 are more specific tumor targeting agents than ^99m^Tc-BN1, ^99m^Tc-BN2, and ^99m^Tc-BNS. As a whole, ^99m^Tc-BN4 has emerged out as the most promising GRPR-positive tumor imaging probe.

## Discussion

Studies involving receptor-targeting radiolabeled bombesin (BN) peptides as radiopharmaceuticals in breast cancer imaging are worth investigating, through scintigraphic studies as non-invasive diagnosis of GRP receptor-positive breast tumors [3, 30, 37, 40, 41]. Rapid degradation of the BN(7-14) fragment instigates the scientists to synthesize newer analogs with some modifications in the natural sequences in order to increase the in vivo stability and affinity. In order to attain high tumor uptake, the peptide should have long survival time in plasma, since it has to be transported from the injection site to the tumor. Besides, it should bind with high affinity to the specific receptor of the tumor tissues to show better uptake in the tumor than the healthy tissues. With this intention, truncated bombesin chain was modified to obtain a reduced speed of its metabolism with retained affinity. This work was focused on the synthesis and preclinical assessment of bifunctional-ligand (HYNIC/DOMA) conjugated four new BN analogs radiolabeled with ^99m^Tc.

During the synthesis of new BN analogs, HYNIC chelator was attached via spacers Asp, Pro, Asn for BN1, BN2, BN3 analogs consequently and DOMA chelator via a spacer GABA for BN4 analog to the N-terminus of BN(7-14)NH_2_ peptide. Besides, non-natural amino acids were introduced, d-amino acids and bulky side chain such as cyclohexyl-alanine (CHAla) by replacing a standard amino acid of the sequences of BN(7-14)NH_2_. Tricine and EDDA co-ligand together have been used for ^99m^Tc labeling because it is expected to form more symmetrical and stable complexes with technetium that permit the control of the hydrophilicity and pharmacokinetics of the labeled peptides [4, 24–27, 39]. The C-terminal of BN(7-14) sequence (Gln^7^-Trp^8^-Ala^9^-Val^10^-Gly^11^-His^12^-Leu^13^-Met^14^-NH_2_) is involved in binding BN peptide to GRP receptor expressing tumors and are as efficient as the full length bombesin tetradecapeptide. For BN1, substitution of Met by Nle at position 14 and Asp was used as spacer; for BN2, substitution of Phe by d-Tyr at position 13 and Pro, Asp was used as spacer and for BN3, substitution of Gly by d-Lys at position 11 and Leu at position 13 by CHAla, Met at position 14 by Nle and Asp, Asn was used as spacer. In the case of BN4, substitution of Gly by d-Pro at position 11 and Leu at position 13 by d-Tyr, Met at position 14 by Nle and GABA was used as a spacer. All the changes of amino acid sequences on BN analogs were expected to induce the fitting of the BN analogs in the active site of different enzymes. Oxidation of sulfur of Met reduces the affinity of the analog; so the Met moiety was replaced by Nle (sulfur free amino acid, in case of BN1, BN3, BN4) to increase the affinity. A promising result was obtained with the insertion of CHAla (in case of BN3) for substitution of Leu at position 13, namely a good receptor affinity of the analogs and considerable increase of the stability in plasma. Insertion of spacers between the BN(7-14) peptide sequence and bifunctional-ligand HYNIC/DOMA have also shown quite good binding and stability properties and specifically the improved results of biodistribution. Introduction of negatively charged residues close to at their N-terminus (as have been previously reported) favors renal clearance of the radiopeptides [3, 4, 26, 27]. Modification of the polarity can change the pharmacokinetics of biomolecules and have a significant impact on the biodistribution as reported for several radiolabeled peptides. Therefore, new hydrophilic linkers were introduced into the newly synthesized BN analogs to improve the in vivo biodistribution and to increase the tumor to background ratios.

Higher radiochemical purity (> 95%) of the ^99m^Tc-labeled peptides, as obtained for the present set of studies, is important as the presence of unlabeled peptides and free ^99m^Tc can saturate the receptors during in vivo studies. The radiolabeled new analogs exhibited the following stability order: ^99m^Tc-BN4 > ^99m^Tc-BN3 > ^99m^Tc-BN1 > ^99m^Tc-BN2 > ^99m^Tc-BNS up to 6 h in PBS and even in the presence of excess cysteine. Higher plasma stability would translate into a higher amount of intact radiolabeled analogs at the tumor area, increasing the chances of targeting the receptors on the tumor cells surface. For in vivo application, one has to be sure that the radiopeptide analog would remain intact before it arrives at the tumor site. Thus the studies involving the in vitro stability of ^99m^Tc-labeled analogs in saline and rat serum and with an excess amount of cysteine or histidine were assessed. All the new analogs are hydrophilic in nature with low protein binding ability, correlating with increased blood clearance and lower persistent of liver uptake. Among the four new analogs, ^99m^Tc-BN3 and ^99m^Tc-BN4 are more hydrophilic than the other. In order to attain substantial radioactivity in the tumor cells, the binding affinity of the analogs to the receptors should be preserved. The binding affinity of the new analogs to GRP receptors on MDA-MB-231 cells was higher than BNS and all affinity results were in nanomolar range. The analogs with the spacer/BFC-ligand exhibit better affinity than the standard analog BNS without spacer and ligand. The affinity results suggest that BN3 and BN4 have a higher binding affinity (3.96 and 3.15 nmol/L) than BNS (5.89 nmol/L). As a consequence, the BN4 analog with the spacer GABA and DOMA ligand exhibits a positive effect on binding affinity. After binding to the receptors, the radiolabeled analogs undergo rapid and high percentage of internalization (65 to 80%) reaching a maximum at 30 min. On the other hand, BN4 (positive charge) showed higher internalization than others, whereas the internalization of BN2 (with two negative charges) was significantly decreased than the others. Internalization was strongly reduced (< 15%) in the presence of excess cold peptide; the process was receptor-mediated. In vitro internalization studies in MDA-MB-231 cells reflect agonistic behavior of the new conjugates, consistent with the previously reported peptide analogs [4, 25–27, 37]. In vitro studies also indicate that the overall molecular charge has a strong influence on the binding affinity and internalization of radiolabeled new analogs. MDA-MB-231 cell-induced tumor model could successfully be developed in cyclosporine-A-treated mice as evidenced from the histology analysis of tumor, liver, lung, and kidney tissue sections (Fig. 7). Expressions of the GRP proteins on MDA-MB-231 cell-induced breast cancer xenografted tumor lesions were confirmed by immunohistochemistry (IHC) staining (Fig. 7). Besides, the dose-dependent increase in different protein expression was confirmed by Western blot analysis (Fig. 8). Highest upregulation of GRPR, HIF-1α, VEGF, p-p38, and PCNA expression with respect to a higher dose of the BN4 analog was recorded. All these results suggest that for the BN4-treated system, hypoxia is induced leading to more tumor growth and more angiogenesis (VEGF, HIF-1α, and GRPR) and more cell proliferation (PCNA and Ki67) that leads to enhanced metastasis of tumor cells. Moreover, IHC analysis also reveals that BN-4 induces activation of angiogenesis (CD31) and proliferation (Ki67) markers (Fig. 8).

Biodistribution studies, performed in breast tumor-bearing mice using these new ^99m^Tc-labeled BN analogs, showed significant uptake in GRP receptor-specific tumor and pancreas and kidney (Table 2 and Fig. 9). It was found that bulk of the radiopeptide was excreted rapidly via the renal route with little radioactivity accumulation in the blood, liver, and muscle. ^99m^Tc-BN4 gets cleared more rapidly from background tissues, presumably as a result of its highest hydrophilicity (Fig. 9). Tumor uptake was highest at 30 min p.i. with a steady decrease over 2-h study periods. While general biodistribution pattern seems very similar for the new ^99m^Tc-labeled BN analogs, few significant differences can be pointed out. Firstly, during the initial time intervals (5 min to 60 min p.i.), ^99m^Tc-BN4 and ^99m^Tc-BN3 showed high and significantly higher uptake than ^99m^Tc-BN1, ^99m^Tc-BN2, and ^99m^Tc-BS in the tumor and the GRP-positive tissues, like the pancreas and intestinal tract. In contrast, at 2 h p.i. radioactivity of ^99m^Tc-BN4 and ^99m^Tc-BN3 were washed out significantly much faster from these tissues compared to the other three analogs (^99m^Tc-BN1, ^99m^Tc-BN2, and ^99m^Tc-BNS). As a result, in the case of ^99m^Tc-BN4 and ^99m^Tc-BN3, high target-to-background ratios and high tumor-to-kidney ratios, as characteristically shown for the 2-h time interval, was found in Fig. 10. The radiotracer uptake decreased in the tumor, liver, stomach, and intestine in the presence of excess cold BNS, indicating the uptake to be receptor-specific whereby the GRP receptors are also expressed in these organs. Presence of a spacer had greater influence in pancreas uptake and tumor uptake, leading to the better tumor-to-background ratio (Fig. 10). Higher receptor density in pancreas compared to tumor would entail the higher uptake found in the pancreas in the in vivo experiments, which has also been reported for other bombesin analogs. Clearance from the blood and normal GRP receptor-negative tissues were very fast for both the analogs ^99m^Tc-BN3 and ^99m^Tc-BN4 which reduce the radiopeptides in circulation and non-target tissues to a minimum. Quantification of uptake in tumor and muscle allowed calculation of tumor to muscle ratios, which is ideally high and increase over time, suggesting high retention in tumor and declining background activity. These new radiopeptides exhibit improved pharmacokinetic and biological activities than BNS and thus they can be used as tumor imaging agents. Moreover, planar imaging studies on breast tumor-bearing mice have been carried out using these new bombesin radiopeptides and showed high tumor uptake and rapid clearance through kidney (Figs. 11 and 12). These new radioligands have shown the advantages of a high level of stability, high binding affinity, rapid localization in its target, and fast clearance from the body. The wide availability of ^99m^Tc was advantageous in preparing these ^99m^Tc-labeled radiopharmaceuticals considered as new tracers for the diagnostic imaging of GRP receptor-positive breast tumors. Among the new HYNIC conjugated bombesin analogs ^99m^Tc-BN3 and DOMA conjugated analogs, ^99m^Tc-BN4 exhibit higher metabolic stability and tumor uptake, and have favorable characteristics for tumor imaging. Finally, the tumor was easily detectable by means of planar imaging with ^99m^Tc-BN4, ^99m^Tc-BN3. This also state that in the comparison between all newly developed radiopharmaceuticals, ^99m^Tc-BN4 provided the best image of the tumor with planar studies than other analogs and have promising characteristics for the detection of GRP receptor-positive tumor that would eventually act as early diagnostic probe for breast tumor imaging. The results encourage the development of new receptor-specific peptides and evaluate its potential as therapeutic radiopharmaceuticals which are considered as the future perspective.

## Conclusions

Four new ^99m^Tc-labeled HYNIC and DOMA conjugated bombesin peptide analogs, ^99m^Tc-HYNIC-Asp-[Phe^13^Nle^14^]BN(7-14)NH_2_: ^99m^Tc-BN1; ^99m^Tc-HYNIC-Pro-Asp-[Tyr^13^Met^14^]BN(7-14)NH_2_: ^99m^Tc-BN2; ^99m^Tc-HYNIC-Asp-Asn-[Lys^11^CHAla^13^Nle^14^]-BN(7-14)NH_2_: ^99m^Tc-BN3; and ^99m^Tc-DOMA-GABA-[Pro^11^Tyr^13^Nle^14^]-BN(7-14)NH_2_: ^99m^Tc-BN4 were synthesized, characterized, and compared with the bombesin ^99m^Tc-BN(7-14)NH_2_: ^99m^Tc-BNS. In vitro and in vivo studies as receptor-argeted tumor imaging probes suggest that the BN analogs can have potentials in tumor diagnosis. The radioligands were more stable, higher binding affinities with rapid localization in target tissues, and fast clearance from the non-target tissues. Among the four radiopharmaceuticals, ^99m^Tc-BN3 and ^99m^Tc-BN4 exhibit superior pharmacokinetic characteristics for scintigraphic detection of GRP receptor-positive tumor. The DOMA ligand displays a profound impact on the binding affinity of the ^99m^Tc-BN4, providing new insights into the design of bombesin analogs. BN4 also stimulates tumor growth and expression of angiogenic markers. The overall results suggest that ^99m^Tc-BN4 possesses optimum properties, thus rendering its potential for non-invasive receptor targeted imaging of tumor, encouraging the researchers to carry out further experiments. Clinical studies and therapeutic effect of these peptide-based radiopharmaceuticals are under process, considered as the future perspectives for theranostic applications in nuclear oncology.

## Additional file

Additional file 1: **Figure S1.** Chemical structure and MALDI mass of new bombesin peptide analogs. A, HYNIC-Asp-[D-Phe^13^Nle^14^]BN(7–14)NH_2_ (BN1); B, HYNIC-Pro-Asp-[D-Tyr^13^Met^14^]BN(7–14)NH_2_ (BN2) and C, HYNIC-Asp-Asn-[Lys^5^-D-CHAla^13^ Nle^14^]BN(7–14)NH_2_ (BN3). **Figure S2.** Chemical structure of ^99m^Tc labeled BN1 (A); BN2 (B) and BN3 (C) with the MM2 energy minimized probable molecular structure of ^99m^Tc-BN1(A’); BN2 (B′) and BN3(C′) (PPTX 3361 kb)

## Abbreviations

^99m^Tc: Technetium-99m
BN: Bombesin
DIPEA: Diisopropyl ethylamine
DMF: Dimethyl formamide
DOMA: 1,4,7-Tri-Boc-10-(carboxymethyl)-1,4,7,10-tetraazocyclododecane-1-yl-monoacetic acid
DOTA: 1,4,7,10-Tetraazacyclododecane-1,4,7,10-tetraacetic acid
EDDA: Ethylenediaminediacetic acid
Fmoc: 9-Fluorenylmethyloxycarbonyl
GRP: Gastrin-releasing peptide
GRPR: Gastrin-releasing peptide receptor
HYNIC: Hydrazinonicotinic acid
SD: Standard deviation
SPECT: Single-photon emission computed tomography
TBTU: [2-(1H-Benzotriazol-1-yl)-1,1,3,3-tetramethyluroniumtetramethyluronium tetrafluoroborate
