# Supplementary figures and images for: HYNIC and DOMA conjugated radiolabeled bombesin analogs as receptor-targeted probes for scintigraphic detection of breast tumor
Source: EJNMMI Res. 2019 Mar 18;9:25. doi: 10.1186/s13550-019-0493-x (PMC6423188; doi:10.1186/s13550-019-0493-x)

## Slide 1
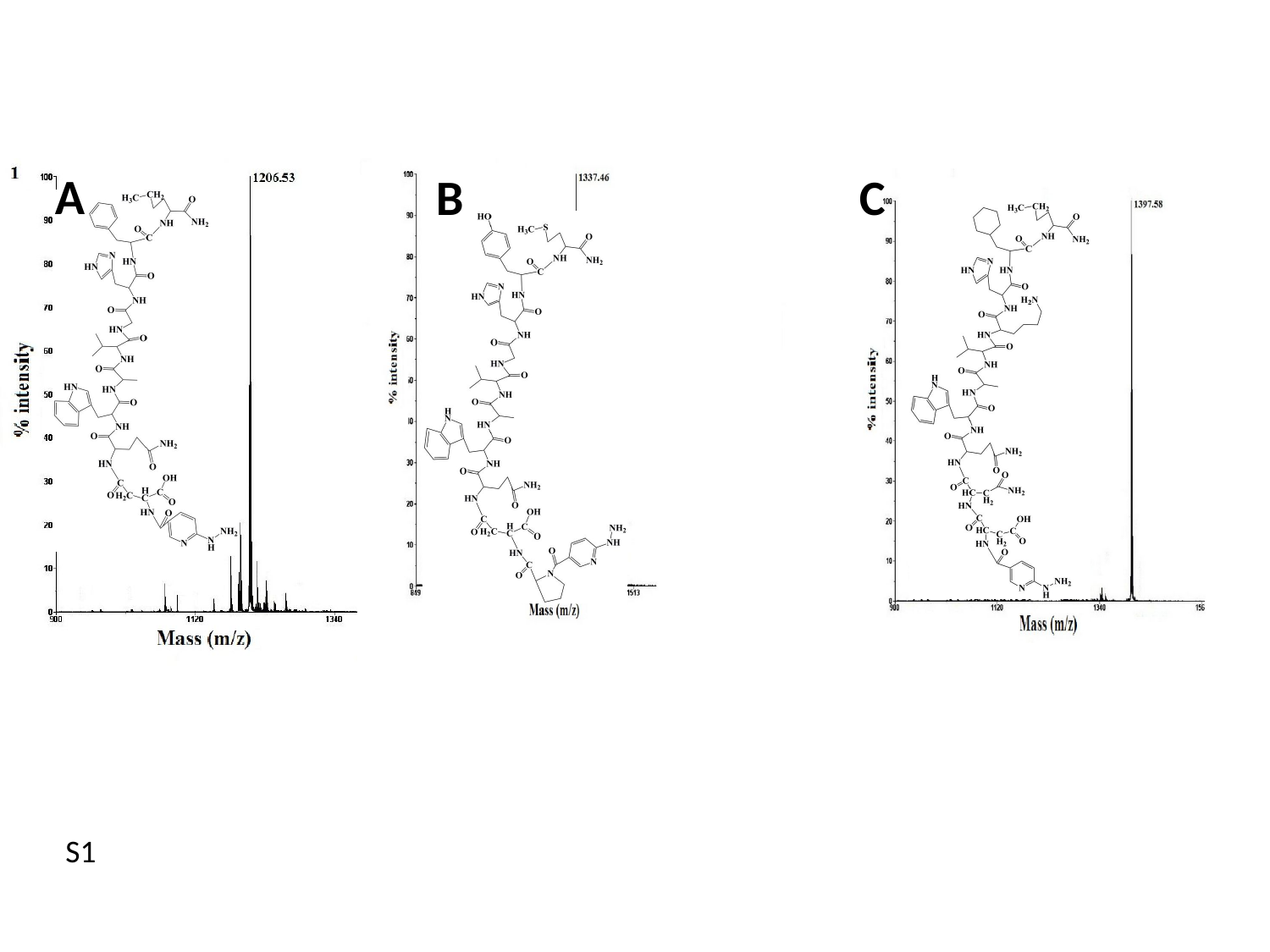

A
B
C
S1

## Slide 2
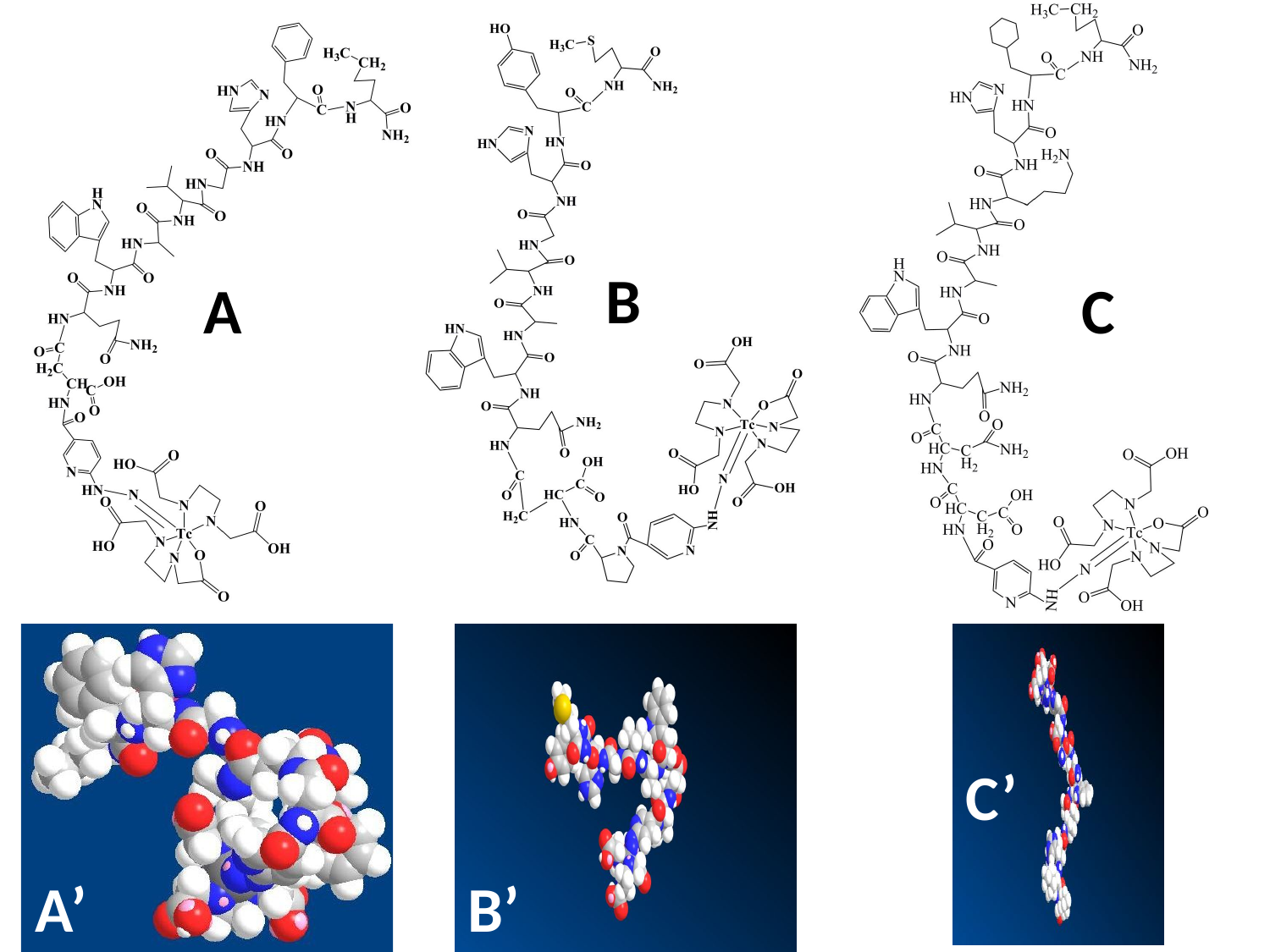

B
A
C
C’
A’
B’

Supplement: Supplementary file 1 — Figure S1. Chemical structure and MALDI mass of new bombesin peptide analogs. A, HYNIC-Asp-[D-Phe13Nle14]BN(7–14)NH2 (BN1); B, HYNIC-Pro-Asp-[D-Tyr13Met14]BN(7–14)NH2 (BN2) and C, HYNIC-Asp-Asn-[Lys5-D-CHAla13 Nle14]BN(7–14)NH2 (BN3). Figure S2. Chemical structure of 99mTc labeled BN1 (A); BN2 (B) and BN3 (C) with the MM2 energy minimized probable molecular structure of 99mTc-BN1(A’); BN2 (B′) and BN3(C′) (PPTX 3361 kb) [file 13550_2019_493_MOESM1_ESM.pptx]
